# Unveiling Epigenetic Regulatory Elements Associated with Breast Cancer Development

**DOI:** 10.3390/ijms26146558

**Published:** 2025-07-08

**Authors:** Marta Jardanowska-Kotuniak, Michał Dramiński, Michal Wlasnowolski, Marcin Łapiński, Kaustav Sengupta, Abhishek Agarwal, Adam Filip, Nimisha Ghosh, Vera Pancaldi, Marcin Grynberg, Indrajit Saha, Dariusz Plewczynski, Michał J. Dąbrowski

**Affiliations:** 1 Computational Biology Group, Institute of Computer Science of the Polish Academy of Sciences, 01-248 Warsaw, Poland; marta.jardanowska@ipipan.waw.pl (M.J.-K.); m.draminski@ipipan.waw.pl (M.D.);; 2 Institute of Biochemistry and Biophysics of the Polish Academy of Sciences, 02-106 Warsaw, Poland; greenb@ibb.waw.pl; 3 Laboratory of Bioinformatics and Computational Genomics, Faculty of Mathematics and Information Science, Warsaw University of Technology, 00-662 Warsaw, Poland; michal.wlasnowolski@pw.edu.pl (M.W.); k.sengupta@datascience.edu.pl (K.S.); 4 Laboratory of Functional and Structural Genomics, Centre of New Technologies, University of Warsaw, 02-097 Warsaw, Poland; a.agarwal@cent.uw.edu.pl; 5 Department of Computer Science and Engineering, Shiv Nadar University, Chennai 201314, India; nimishaghosh@snuchennai.edu.in; 6 Cancer Research Center Toulouse, National Centre for Scientific Research (CNRS), Inserm, Université de Toulouse, 31037 Toulouse, France; vera.pancaldi@inserm.fr; 7 Department of Computer Science and Engineering, National Institute of Technical Teachers’ Training and Research, Kolkata 700106, India; indrajit@nitttrkol.ac.in

**Keywords:** breast cancer, chromatin structure, epigenetic regulation, MCFS-ID, Monte Carlo Feature Selection, Natural Language Processing, *NKAPL*, NRF1, transcription factor

## Abstract

Breast cancer affects over 2 million women annually and results in 650,000 deaths. This study aimed to identify epigenetic mechanisms impacting breast cancer-related gene expression, discover potential biomarkers, and present a novel approach integrating feature selection, Natural Language Processing, and 3D chromatin structure analysis. We used The Cancer Genome Atlas database with over 800 samples and multi-omics datasets (mRNA, miRNA, DNA methylation) to select 2701 features statistically significant in cancer versus control samples, from an initial 417,486, using the Monte Carlo Feature Selection and Interdependency Discovery algorithm. Classification of cancer vs. control samples on the selected features returned very high accuracy, depending on feature-type and classifier. The cancer samples generally showed lower expression of differentially expressed genes (DEGs) and increased *β*-values of differentially methylated sites (DMSs). We identified mRNAs whose expression is explained by miRNA expression and *β*-values of DMSs. We recognized DMSs affecting NRF1 and MXI1 transcription factors binding, causing a disturbance in *NKAPL* and *PITX1* expression, respectively. Our 3D models showed more loosely packed chromatin in cancer. This study highlights numerous possible regulatory dependencies, and the presented bioinformatic approach provides a robust framework for data dimensionality reduction, enabling the identification of key features for further experimental validation.

## 1. Introduction

In 2021, the WHO announced that, for the first time in 20 years, the most commonly diagnosed cancer in the world was not lung cancer but breast cancer. According to the GLOBOCAN report publishing cancer statistics for 2020 based on data from 185 countries and 36 different types of cancer, 2.3 million people were affected by breast cancer and 684,996 people died from it [1]. It means that, currently, almost one in four oncology female patients develops breast cancer. Early-stage cancer detection is crucial to apply the most effective therapy available [2]. Due to the heterogeneous nature of breast cancer and the large amount of information to be considered, the implementation of appropriate treatment is extremely difficult [3].

The effectiveness of cancer therapies is closely related to diagnostic accuracy. Inclusion of molecular features in the classification of cancers [4,5] has allowed for the development of targeted treatment. Further molecular studies aimed at detecting cancer markers and potential drug targets are essential to further improve available therapies. Large molecular databases, while extremely rich in information, are also burdened with a certain level of information noise, which is a significant analytical challenge, especially when the number of samples available is limited and data dimension is high. In such a case, the risk of obtaining false positives and false negatives may be higher because, from the statistical perspective, the problem is ill-defined [6]. To overcome this challenge, in this research, we applied the Monte Carlo Feature Selection and Interdependency Discovery (MCFS-ID) algorithm [7] to reveal significant signals related to breast cancer in various molecular datasets [8]. The MCFS-ID has been successfully used in a broad range of scientific disciplines, including oncology, virology, and cardiology [9,10,11,12,13].

It is a known fact that breast cancer is associated with multiple DNA mutations and genome rearrangements that affect cell physiology, resulting in gene expression changes [14,15]. Yet, it has been shown that DNA alterations alone cannot fully explain breast cancer development, and in recent years there has been accelerated research toward the epigenetic regulation of cancer-related gene expression [16,17,18,19,20].

One of the most important and well-studied epigenetic modifications is DNA methylation. Methylation of gene promoters and regulatory regions plays an essential role in regulating gene expression and shows high variation across cell types. Its deregulation is associated with tumorigenesis [21,22,23] and was demonstrated to have a role in predicting patient survival [17]. The DNA binding affinity of multiple transcription factors (TFs) relies on DNA methylation patterns [24,25]. Interestingly, DNA hypo-methylation is present in the regulatory regions of oncogenes promoting tumorigenesis [26,27,28], while hyper-methylation is frequently connected with the silencing of tumor suppressor genes [29,30,31], but other research shows that these patterns may be more complex and depend on genomic location of the methylation alterations [32,33]. That is why a further large-scale analysis of locus-specific DNA methylation patterns in relation to TF affinity and the level of gene expression may bring novel knowledge about cancer biomarkers and deregulated biological pathways that promote tumorigenesis.

Similarly to DNA methylation, miRNA can also act as an epigenetic regulator [34,35] that may post-transcriptionally modulate breast cancer related mRNA genes affecting its development and drug resistance of breast cancer [36,37]. Another epigenetic mechanism known to contribute to cancerogenesis is the 3D chromatin structure alterations, which can be affected by several molecular elements including point mutations [38] or DNA methylation levels [39]. It was shown to disrupt gene expression in many cancers [40,41]. Therefore, defining the distances between regulatory elements and their target promoters in 3D chromatin structure may provide insights into the underlying mechanisms of genomic regulation also in breast cancer.

In the present study, we applied the MCFS-ID algorithm to extract the significant transcriptomic and DNA methylation features from The Cancer Genome Atlas (TCGA) dataset that could distinguish between healthy and cancerous tissues. Subsequently, we conducted analyses of mRNA expression, DNA methylation, detection of TF motifs, miRNA potential targeting by drugs, and modeling of 3D chromatin structure. This integrative approach helped reveal the biological importance of the selected features as well as the direct and indirect connections between them and their impact on the initiation and development of breast cancer.

## 2. Results

### 2.1. Detection of Potential Breast Cancer Biomarkers Using the MCFS-ID Algorithm

Our study aimed to verify if there are significant molecular features and interactions between them that may be important for breast cancer prediction and possibly used as biomarkers. The Monte Carlo Feature Selection and Interdependency Discovery (MCFS-ID) algorithm was used to select top significant features that distinguish cancerous from normal tissue samples from TCGA data. The final feature set was derived by merging MCFS-ID outcomes from two independent steps (see Section 4.2). Firstly MCFS-ID was performed on the joined set consisting of mRNA and miRNA expression and DNA methylation. As a result, the feature ranking was dominated by the methylation features, followed by mRNA expression (Table 1, Table S1). Only six miRNA expression features were found to be relevant in breast cancer prediction. In the next step, three more MCFS-ID experiments were conducted on datasets consisting of single-feature types to verify whether each of those was informative in distinguishing cancer from normal samples (Table S1). With this approach, it was possible to expand the number of significant features, especially for miRNA data, and confirm the statistical significance of each individual set of attributes in sample classification. Finally, out of 417,486 multi-omics input features, 2701 (2006 + 590 + 105) (Table 1) were selected by the algorithm as features potentially involved in cell physiological changes resulting in breast cancer development. The last two columns in Table 1 show significantly high weighted predictive accuracy (wAcc) of support vector machines (SVM) and random forest (RF) models, for classification of samples into cancer vs. normal, where the test samples were not used in the feature selection phase (see Section 4.1). It is worth underlining that after the MCFS-ID run, each feature is evaluated by the RI (relative importance) value so that for each data type, instead of an unordered set of features, a ranking of the most informative features is produced (Table S1).

### 2.2. Descriptive Analysis of mRNAs Having a Significant Predictive Value

The Machine Learning feature selection process focuses on the selection of features based on their high statistical significance. However, it does not consider their biological meaning, which must be examined afterwards. This section provides a detailed biological interpretation and literature-based validation of the identified significant mRNA features. At first, the top 10 mRNA genes from the MCFS-ID ranking (*ADAMTS5*, *COL10A1*, *TMEM220*, *ARHGAP20*, *MMP11*, *CAVIN2*, *PLPP3*, *MICU3*, *MME*, *CD300LG*) were screened, and all of them were confirmed to have an association to cancer prediction and development. The top five were reported as effective cancerous tissue markers [42]. There is a number of scientific research for each of the top 10 mRNA genes, well-documenting their significance and association with cancerogenesis: *ADAMTS5* [43], *TMEM220* [44], *ARHGAP20* [45], *MICU3* [46]; or precisely with breast cancer: *COL10A1* [47], *MMP11* [48], *CAVIN2* (formerly known as *SDPR*) [49], *PLPP3* [50], *MME* [51], and *CD300LG* [52]. They confirm the potential usefulness of the implemented approach. These findings strongly support the potential utility of these identified mRNA genes as promising biomarkers for breast cancer detection and progression monitoring, warranting further clinical validation.

**Figure 1:**
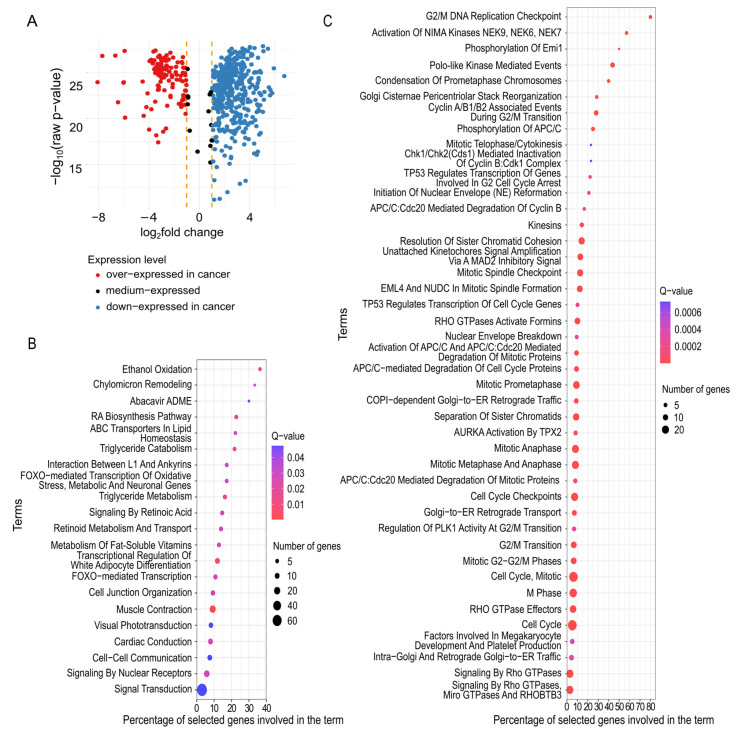
Overview of mRNAs indicated as significant in distinguishing cancer from normal tissue samples. (**A**) The volcano plot shows differences in the expression levels of 590 mRNA considered significant in the cancer/normal prediction in the feature selection set (adjusted raw *p*-value < 0.05 and log_2_FC = ±1). (**B**) Enriched pathways from the Reactome pathway database for down-regulated genes. (**C**) Enriched pathways from the Reactome pathway database for over-expressed genes. To allow for better readability, the number of less enriched pathways in the graph was reduced with the cutoff q-value = 0.001 (all terms for cutoff q-value = 0.05 are available in Table S2).

Subsequent analysis included all significant mRNA genes to unveil their biological role in cancerogenesis and to discover new significant bio-functional relationships. Among the 590 mRNA genes returned by MCFS-ID, 576 revealed differential expression when filtering by the required log_2_FC and adjusted *p*-value (Figure 1A). Interestingly, these differentially expressed genes (DEGs) seem to be strong, independent predictors of breast cancer. At the level of feature selection performed with MCFS-ID, the returned decision trees (see Section 4.2) presented very shallow depth; classical statistical tests confirmed that these genes significantly differed in expression between cancer and normal samples. Moreover, the majority of DEGs demonstrated a lowered expression in cancer (*n* = 447), whereas only 129 showed increased expression. The down-expressed genes were enriched in 16 pathways from the Reactome database [53], which showed a great functional heterogeneity (Figure 1B). Over-expressed genes were enriched in 91 pathways (Figure 1C and Table S2), and most of them were related to the cell cycle and mitosis. Down-expressed DEGs were enriched in pathways related to lipids regulation and transport, neurotransmission, and retinoic acid synthesis (Figure 1B). These findings were confirmed by a Natural Language Processing approach (NLP). The pathways in which DEGs were enriched correspond very well to the unique keywords that describe clusters built on the gene function descriptions using NLP methods and hierarchical clustering (see Section 4.3). The two most numerous clusters (mostly down-expressed) were related to the following terms: ‘regulation and metabolic processes’ and ‘ion transmembrane transport’ (Table 2). Finally, we confirmed that the set of 590 mRNA genes was significantly enriched in genes-related (*n* = 79) and immunological processes (chi-squared test, *p* < 0.05). This fact confirms the well-known engagement of immune-related genes in cancerogenesis.

### 2.3. Genomic Context of DNA Methylations with Predictive Value

Feature selection revealed 2006 significant sites that differed in methylation levels between cancer and normal samples (Table 1), hereafter called differentially methylated sites (DMSs). Out of all DMSs, only one locus (cg02025583), located within the promoter of one of the top 10 mRNA genes, returned in the main MCFS-ID ranking. This promoter precedes the *TMEM220* gene, and the cytosine cg02025583 is overlapped by a motif of the E2F2 transcription factor (TF), which is a good example of an altered epigenetic regulation of gene expression.

**Figure 2:**
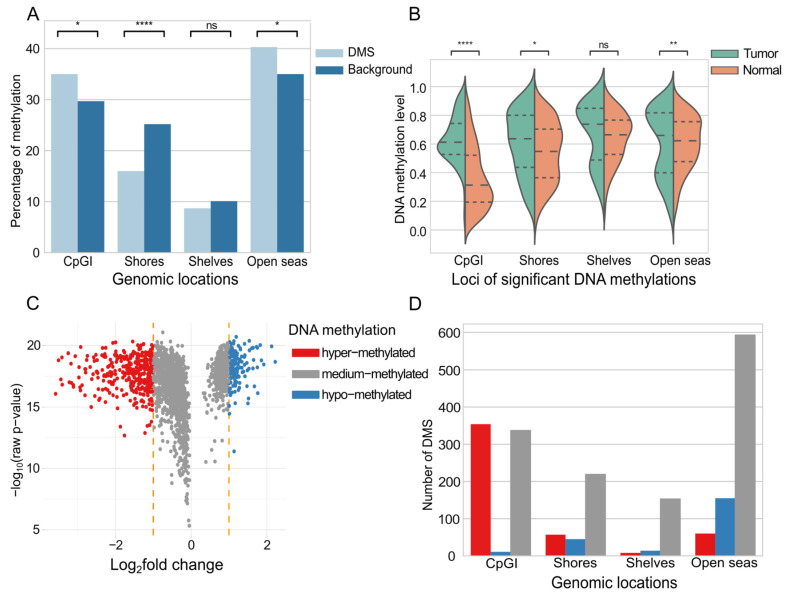
Characteristics of 2006 significant DNA methylation sites (DMSs). (**A**) Distribution of loci included in the Illumina 450K array in comparison to the distribution of DMSs. (**B**) DMSs’ *β*-values distribution in the tumor and normal samples with respect to the specific genomic regions. (**C**) DMSs assigned to hyper/medium/hypo-methylated with respect to log2 Fold Change in β-values. (**D**) Number of loci with hyper/medium/hypo-methylated DNA in particular types of genomic regions. Significance coding used throughout the panels (**A**) and (**B**): ns *p* > 0.05 (not significant); * *p* ≤ 0.05; ** *p* ≤ 0.01; **** *p* ≤ 0.0001.

To assess if the distribution of significant DMSs was random, we compared it to the background distribution of all CpG probes on the Illumina 450K array. This revealed a statistically significant, non-random distribution of DMSs across genomic regions, with enrichment in CpG Islands (CpGI) and open seas and depletion in shores (chi-squared test, corrected *p*-value ≤ 0.05, Figure 2A). The indicated sites are candidates for modulating activity of regulatory regions; therefore, we focused on their methylation levels in normal vs. cancer samples to unveil their putative regulatory role in cancer development. We found that DMSs’ methylation *β*-values showed a significant shift towards higher values within CpGI and presented significantly different distribution of *β*-values within shores and open seas between cancer vs. normal samples (Wilcoxon test *p*-value ≤ 0.05, Figure 2B). There were over two times more hyper-methylated DMSs (*n* = 479) than hypo-methylated DMSs (*n* = 225) discovered in tumors (Figure 2C). Interestingly, the vast majority of hyper-methylated DMSs were located within CpGI, which are well-known gene transcription regulators (Figure 2D). Medium-methylated DMSs showed very high frequency not only in CpGI but also in shores, shelves, and open seas (Figure 2D).

To investigate a potential regulatory association of gene expression mediated by DNA methylation, the Spearman correlation was measured between mRNA levels of 590 significant genes and *β*-values of each DMS within 1 Mbp upstream and downstream from TSS of these genes. The correlation cut-off value was set to |*rho*| ≥ 0.6, and there were 59 pairs meeting this condition (Table S3). The majority of the obtained correlations were negative (*n* = 44), with only a few positive (*n* = 15). A more frequent negative correlation was expected if DNA methylation located in promoters or enhancers inhibited gene expression. Among these pairs, there were 34 unique genes and 55 unique DMS loci. Almost all genes were down-regulated in tumor samples, but the *HN1L* and *KIFC1* were up-regulated. Out of 55 DMSs, five were located within one gene promoter. Interestingly, all of them were hyper-methylated and were close to each other, within one CpGI in a range of 25 bp and within the *NKAPL* gene promoter (Figure S1). Two of them (cg18694169 and cg10253847) were overlapped by a motif of the NRF1 TF. NRF1 normally activates gene expression, but here, due to hypermethylation, its binding to DNA can be inhibited by decreased affinity or blocked if MBD protein binds at this site, which may depend on the CpG density. Accordingly, *NKAPL* was down-regulated in cancer samples, which could be explained by hyper-methylation of the five DMSs [54]. The other 50 DMSs were not assigned to any promoter and also had a confirmed chromatin state, indicating possible activity (Table S3); therefore, they were defined as potential distal regulatory factors.

**Figure 3:**
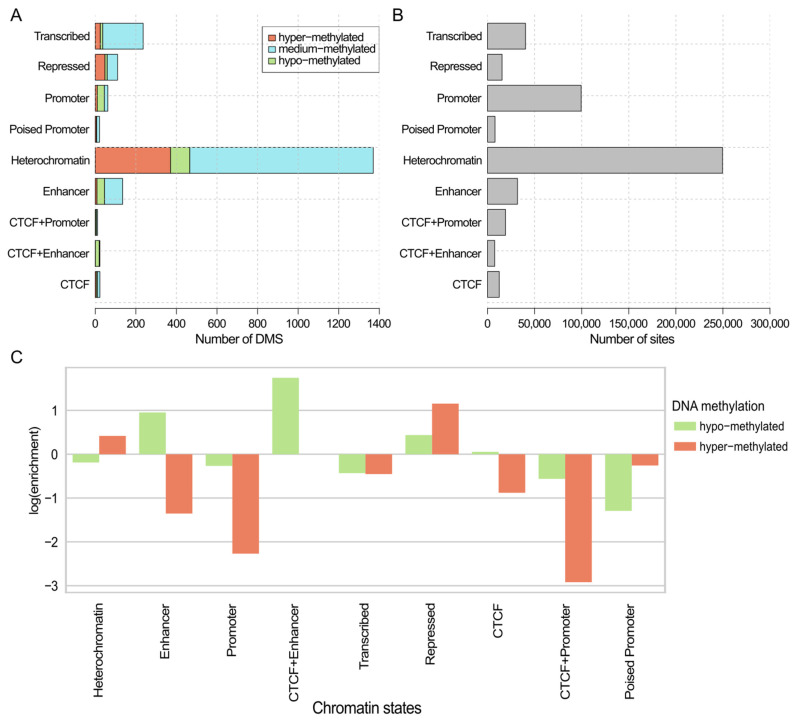
Distribution of cytosines across chromatin states obtained for the MCF-7 breast cancer cell line. (**A**) Number of DMS in individual chromatin states. (**B**) Illumina 450K sites assigned to individual chromatin states, representing the background distribution. (**C**) Differential distribution of hypo- and hyper-methylated DMS in specific chromatin states.

In order to have a better understanding of the potential role of the DMSs in gene expression regulation, all DMSs were intersected with the chromatin states of MCF-7 breast cancer cell line (Figure 3A); simultaneously, all sites from Illumina 450K were intersected with the chromatin states of MCF-7 as well (Figure 3B). The observed distribution of DMSs across chromatin states was similar to the distribution of all Illumina 450K sites across the chromatin states (chi-squared test *p*-value > 0.2). Comparison of the distribution of hypo- and hyper-methylated DMSs revealed a visible enrichment of hyper-methylated DMSs in transcriptionally inactive chromatin states, such as heterochromatin. At the same time, hyper-methylated DMSs were depleted within states associated with gene transcription activation, such as enhancer, promoter, and transcribed regions (Figure 3C). Conversely, for the hypo-methylated DMSs, the opposite pattern was observed (Figure 3C).

Next, the evaluation of the impact of DMSs on survival was tested. Out of 2006 DMSs there were 691 (Table S4) that were found to be significantly correlated with patients’ survival (*p* < 0.05). The number of loci associated with the survival in 100 random picks of 2006 methylation probes was between 375 and 450, which confirms that MCFS-ID returned an enriched list (Figure S2). However, after application of the correction for multiple tests, none of the sites achieved expected statistical significance. Therefore, the prediction of patients’ survival by the DMSs should be treated with caution.

### 2.4. Biological Role of Significant miRNA Genes

Firstly, for the top 10 miRNAs identified by MCFS-ID, it was confirmed that they are associated with breast cancer biological processes, namely miR-139 [55], miR-10b [56], miR-21 [57], miR-183 [58], miR-145 [59], miR-99a [60], miR-182 [61], miR-96 [62], miR-486 [63], and miR-141 [64].

Next, to characterize the regulatory functions of all 105 miRNAs identified in the MCFS-ID experiment, their associations with mRNA from the miR + Pathway database were verified, resulting in the detection of 822 unique mRNAs linked to significant miRNAs. Out of these mRNAs, 43 were shown to be significant in predicting breast cancer in the MCFS-ID analysis (Table 1). The intersection with the mRNA clusters returned by NLP analysis showed that out of 43 mRNAs, 32 belong to cluster 1, one mRNA to cluster 2, two to cluster 3, seven to cluster 5, and one to cluster 6 (Table 2). Most of the mRNAs assigned to cluster 1 had a decreased expression in the cancer samples, but five had an increased expression. The opposite situation could be observed in cluster 5, where six mRNAs were upregulated in the tumor and one was down-regulated.

For 58 miRNA genes’ down-expressed in cancer, selected out of the 105 significant miRNAs (Figure S3B), the target mRNA genes were assigned to them, and the putative associations between 46 miRNAs and 126 mRNAs were confirmed with Spearman correlation (*rho* ≤ −0.2, Table S5). KEGG pathway analysis of these 126 mRNAs returned insignificant results (adj. *p*-value > 0.05). The mRNAs contributing to significant correlations formed a protein–protein interaction network consisting of 2265 proteins. For the 50 proteins with the highest number of interactions in this network, the miRNAs targeting their mRNAs were assigned (Table S6), resulting in 22 unique miRNAs, all initially obtained as significant in the main MCFS-ID run. KEGG pathway analysis of the genes encoding those 50 proteins showed significant annotations to breast cancer (*p*-value = 0.000454) as well as to many other cancers, e.g., melanoma, renal cell carcinoma, acute myeloid leukemia, and colorectal cancer (Figure S4A). These proteins were also found to be associated with cancerogenesis-related biological processes, e.g., chemical, viral or proteoglycans, as well as pathways known to be crucial for cancer development, e.g., p53 signaling pathway (Figure S4A). The returned terms from GO BP analysis were almost all related directly or indirectly to cell cycle, the crucial process for cancer development and progression (Figure S4B). Moreover, out of the 50 proteins with the highest number of interactions in the miRNA-regulated protein–protein network, 16 are known drug targets in breast cancer treatment. The largest number of them was targeted by palbociclib, ribociclib [65], and abemaciclib [66], among 19 others drugs (Table S7). It is worth mentioning that both scores showed in Table S7, i.e., Drug Score (DScore), which measures suitability of the drug according to the genomic profile, and Gene Score (GScore), which reflects biological relevance of genes in the tumoral process, had high values for the majority of the aforementioned drugs, indicating their significant effect (Table S7). The resulting miRNA–protein–drug network is visualized in Figure S3C. There are two mRNA genes specified in this network (*CDC25A* and *BIRC5*) whose expression significantly correlated with upregulated miRNAs, namely hsa-mir-100 and hsa-mir-218-2, which are also known to be breast cancer drug targets (Figure S4A,B, Table S7). These two miRNAs were selected by MCFS-ID main run.

### 2.5. Detection of miRNA and DNA Methylation Loci Significant in the Context of Predicting mRNA Expression Levels

**Figure 4:**
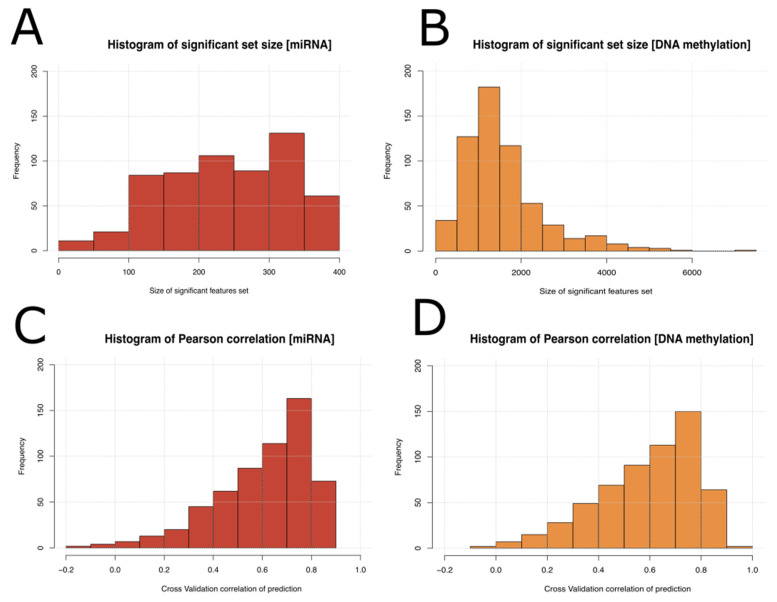
Summary of mass MCFS-ID experiments on 590 mRNA genes. (**A**) Distribution of the number of significant miRNA features returned across 590 experiments. (**B**) Distribution of the number of significant DNA methylation loci returned across 590 experiments. (**C**) Distribution of the Pearson correlations obtained for linear models built on significant miRNA features returned across 590 experiments (**D**) Distribution of the Pearson correlations obtained for linear models built on significant DNA methylations returned across 590 experiments.

The result of 590 MCFS-ID experiments run on 590 significant mRNA features (Table 1), each separately used as the target variable, with miRNA expression or DNA methylation set as predictors, showed that miRNA features are better predictors than methylation. This section further explores the biological relevance of these selected miRNA and DNA methylation loci, especially those with high predictive values across multiple-target mRNAs. Out of 590 mRNA expression features, only 73 could be correctly predicted by miRNA features, 66 by DNA methylation features, and 39 by both (where Pearson correlation level ≥ 0.8). For each target variable (out of 590), a different significant set of features was returned. These sets differed in size and contained different features in the top ranking; therefore, it was possible to analyze the distribution of significant set size (based on MCFS-ID cutoff), Pearson correlation calculated between each mRNA expression, and its prediction based on the significant feature set. To find out if, for a different mRNA, there were common predictive miRNAs or DNA methylations, the frequency of a single significant feature across all significant feature sets was calculated as well (separate for miRNA and DNA methylation). Histograms show that the number of selected significant DNA methylations in the rankings was much greater than for the miRNA (Figure 4A,B), which may correspond to the size of the input datasets and to the fact that statistical modeling of the mRNA expression is much more complex in the case of DNA methylation data. However, the quality of the prediction of mRNA expression, based on miRNA features (Figure 4C,D), is comparable to that achieved with the help of DNA methylation data.

To obtain a better overview of all the selected top features, all MCFS-ID top rankings (separated by data category) were combined, and, for each predictor feature, the sums of RI, mean RI, and frequency (how many times a single feature was found as significant) were calculated. The resulting two rankings—separate for miRNA expression and DNA methylation—are shown in Table 3 (the top 15 features) and the Supplementary Material (Tables S8 and S9). For 73 mRNA target features (that could be predicted with Pearson correlation level ≥ 0.8), 97 miRNA predictors were found by MCFS-ID as significant (Freq column), which means that the same miRNAs took part in a successful prediction of the 73 protein coding genes’ expression. The predictive impact of particular miRNAs depended on mRNA, which was observed by a different position in a single MCFS-ID ranking. Moreover, 81 out of 97 miRNA genes were also significant in cancer prediction and selected by the main MCFS-ID experiment (see Section 2.1 and Table 1). The columns ‘Sum RI’ and ‘Mean RI’ in Table 3 accumulate the RI (Relative Importance) values from all MCFS-ID experiments where correlation level was ≥0.8. The last column refers to the ranking of the main MCFS-ID experiment (on a given data type). Additionally, all the top-15 miRNA genes in the table are confirmed as cancer-specific in the literature according to www.mirbase.org (accessed on 1 March 2023).

### 2.6. Tracking Associations Between DMSs and Detected TF Motifs

Depending on the cytosine location in the genome or changes in its DNA methylation level, the cytosine loci overlapping transcription factor binding sites (TFBS) may significantly affect binding affinity of a TF to the DNA. To investigate this issue, for each DMS, a DNA sequence covering 41 bp was obtained (site +/− 20 bp), and the TF motif search was applied. First, DMSs were divided according to their location in genomic regions: promoters, gene bodies, and intergenic. There were 616 DMSs located within promoters, 1037 in gene bodies, and 353 in intergenic regions, and the motif search returned 48, 54, and 21 TF motifs in these three genomic regions, respectively. The numbers of returned TF motifs proportional to DMSs reflect no significant enrichment in the mentioned specific genomic regions (chi-squared test, *p*-value = 0.138). Moreover, it was confirmed that none of the protein families were overrepresented among the confirmed motifs within the three genomic regions (*p*-value after FDR correction > 0.05). There were 45 common motifs between promoter and gene body regions, of which 21 were also shared with the intergenic. Specifically, we detected three TFs (E2F1, ESR2, and NRF1) only in the promoters and nine (ELK3, HIF1A, ITF2, LYL1, NFIA, NR2C2, SNAI1, ZN341, and ZN589) only within the gene bodies (Table S10).

**Figure 5:**
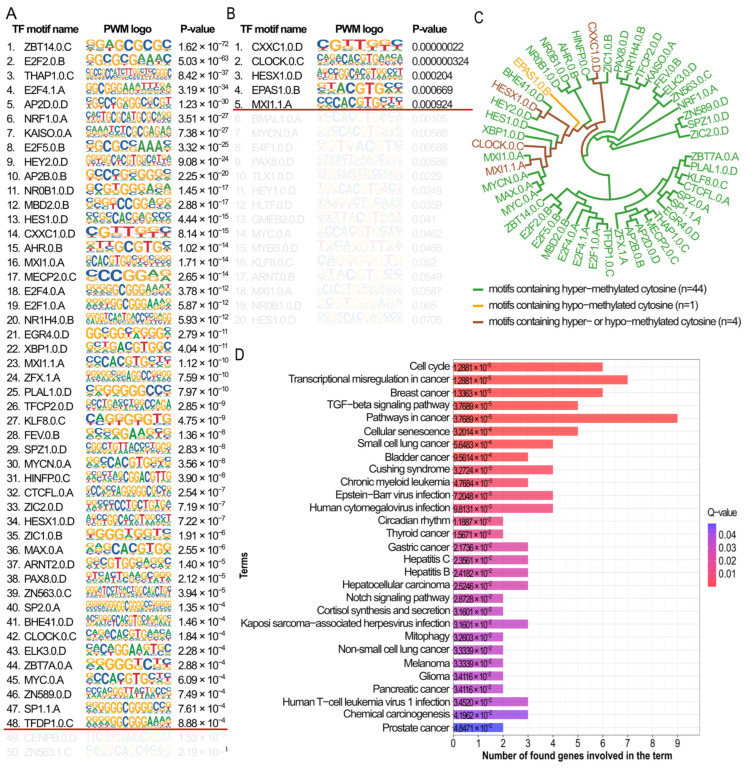
TF motifs overlapping differentially methylated cytosines. (**A**) TF motifs overlapping hyper-methylated DMS. (**B**) TF motifs overlapping hypo-methylated DMS. In (**A**,**B**), the red horizontal line indicates the *p*-value cut-off point. (**C**) Hierarchical clustering of TF motifs based on their PWMs. (**D**) Functional analysis of genes encoding TFs whose motifs overlapped DMSs hyper-methylated in cancer (KEGG database). The list of genes related to specific terms is shown in Table S11.

When verifying TF motifs overlapping hyper-methylated (*n* = 479) cytosines, we confirmed 48 motifs; for hypo-methylated loci (*n* = 225), there were 5 motifs detected. Only one motif, EPAS1.0.B, was specific to the hypo-methylated set of DMS; the remaining four were detected for both cytosine methylation levels (Figure 5A,B).

To verify the similarities of PWMs of the detected TF motifs (Figure 5A,B), the hierarchical clustering was performed; it showed that the majority of motifs overlapping hyper-methylated cytosines (Figure 5A) constituted two homogenous clusters. The third cluster contained motifs overlapping both hypo- and hyper-methylated cytosines; therefore, it seems that TF motifs have some common characteristics independent from cytosine methylation level. Next, using the KEGG database [67] (Figure 5D), it was verified that the majority of biological pathways related to genes encoding TF motifs overlapping hyper-methylated DMS were precisely connected with cancer (e.g., misregulation in cancer, breast cancer, lung cancer, etc.) as well as through pathways, which are well-known to be connected with tumorigenesis (e.g., TGF-β signaling pathway [68], human cytomegalovirus infection [69]). Of note is the group of genes *SP1*, *MYC*, *HEY2*, *E2F1*, *E2F2*, and *HES1*, which are known to be associated with the KEGG breast cancer functional pathway (Figure 5D).

### 2.7. Models of Regulatory Networks

To discover interdependencies between DMSs and genes encoding TF motifs overlapping these DMSs in the context of breast cancer prediction, another MCFS-ID experiment was conducted (Table S12). Only the genes encoding TFs that were overlapping significant DMSs, as well as the levels of DNA methylations of these DMSs, were considered. The MCFS-ID algorithm returned 281 significant features (Table S13), out of which the vast majority were DMSs with only seven genes encoding TFs, namely *MXI1*, *EPAS1*, *PLAGL1*, *E2F1*, *NR0B1*, *BHLHE41*, and *ARNT2*. These 281 features were annotated to their closest genes resulting in identification of 279 mRNA genes named hereafter target genes. Nine of them (*PITX1*, *TGFBR3*, *PAFAH1B3*, *TAL1*, *TDRD10*, *SHE*, *LEP*, *TMEM220*, and *NKAPL*) were previously reported in the main MCFS-ID ranking (Table S1). Only one target gene, *PITX1*, contained a motif of one of the seven TFs encoded by the significant genes identified in the second MCFS-ID experiment, namely *MXI1*, which was overlapping hyper-methylated DMS cg00396667 cytosine; the remaining eight target genes were linked only to the DMS but not to TFs. For breast cancer, it was confirmed that the down-regulation of *PITX1* improves prognosis, and this gene is associated with DNA methylation levels [51,70].

**Figure 6:**
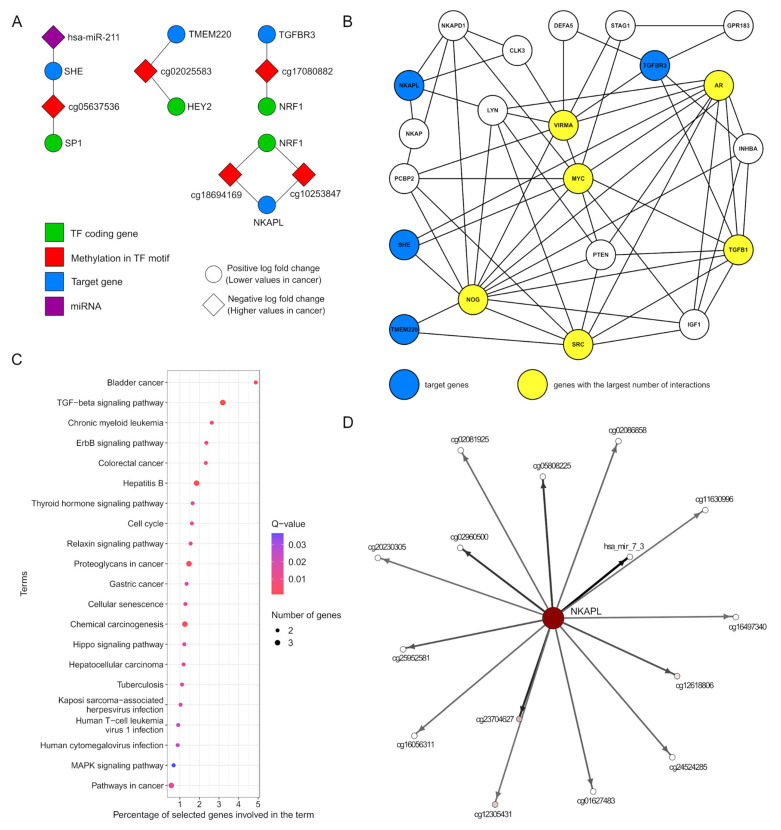
Target genes interactions, biological functions and graphical representation of their putative regulatory elements. (**A**) Visualization of interactions driven from linear models. (**B**) The network of gene–gene interactions created for the identified target genes visualized in (**A**). (**C**) KEGG pathway analysis for 10 genes highlighted in (**B**). (**D**) ID-graph for *NKAPL* gene.

To review possible regulatory dependencies, the mRNA expression values of these nine target genes were used as prediction variables in a set of linear models. As the result, four linear models for the target genes *TMEM220*, *NKAPL*, *TGFBR3*, and *SHE* reached statistical significance (*p* ≤ 0.05, R^2^ > 0.5), and these genes were located on the 3rd, 44th, 245th, and 311th position in the main MCFS-ID ranking, respectively (Table S1). All four linear models were highly impacted by tissue type value; however, after removing this feature from the set of explanatory variables, the prediction of the models held a relatively high level (Pearson correlation calculated between target gene expression and predicted value decreased from 0.7–0.8 to 0.6–0.7 after removing tissue type—see Table S14). This observation suggests that the relation between target gene expression and linked TFs features is noticeably strong and specific in the context of tissue type. The four target genes were down-regulated in the tumor samples, suggesting that they were tumor suppressors regulated by hyper-methylated DMSs that reduced TFs’ binding affinity. Moreover, these DMSs were in heterochromatin regions, which was in line with gene silencing. To illustrate the hypothesis that DMSs located within the TF motif would cause disruption of the TF binding, we visualized features of the linear models in a way that the symbols of genes that encode TFs were first connected with DMSs and then with their target gene (Figure 6A). Additionally, based on the Spearman correlation results between the up-regulated miRNAs and their top target mRNAs (Table S5), one additional association of hsa-miR-211 with *SHE* was detected and added to the visualization (Figure 6A).

Additionally, to obtain a better insight into tumor-suppressive paths involving target genes, the gene–gene interactions were established using Pathway Commons and aggregated into one network (Figure 6B). At first, the direct interactions with a group of new genes were established, and then the most frequent of these new genes were used as an input to Pathway Commons to obtain levels of indirect interactions. This approach helped build a more dense network to select genes with the largest number of putative interactions (*MYC*, *NOG*, *TGFB1*, *VIRMA*, *SRC*, and *AR*) pointing at their significance in this network. Genes *NOG*, *TGFB1*, *VIRMA*, and *SRC* were over-expressed in cancer. Unexpectedly, *MYC* was down-expressed, and *AR* showed no significant difference in the Wilcoxon statistical test (Figure S5A). Genes *MYC* and *AR* are known to be over-expressed in specific breast cancer subtypes [71,72,73], but this pattern was not proven for the entire, much more heterogeneous, cohort of TCGA breast cancer samples.

Biological pathway analysis of the aforementioned six genes and target genes used in the linear models (*TMEM220*, *NKAPL*, *TGFBR3*, and *SHE*) showed enrichment of biological pathways related to various cancer types (e.g., bladder cancer, chronic myeloid leukemia, proteoglycans in cancer) and to signaling pathways whose alterations are often associated with carcinogenesis, e.g., TGF-β-signaling [68], erbB-signaling [74], and relaxin-signaling pathways [75] (Figure 6C).

Finally, the Interdependency Discovery function of the MCFS-ID algorithm was used to find statistically significant, nonlinear interactions between features that amplified each other in the classification task. Figure S6 provides four ID-graphs created for the top50 features and the top 50 strongest links between the features. These figures were created as an additional result of four main MCFS-ID experiments (described in the Section 4.2). Figure 6D shows a part of the ID-graph created for the *NKAPL* gene and its neighbors, connected by edges that represent the power of interaction between them. Recently, this gene has been shown as a significant driver of cancer development [76], a prognostic marker [77], and an important factor associated with resistance to pharmacotherapy [78]. The visible directions of interactions show that the *NKAPL* gene expression plays a major role in the classification of normal and tumor tissues, and the remaining DNA methylation and miRNA features boost its predictive power. Notice that the hsa_mir-7_3 is known as a significant factor contributing to cancerogenesis [79]. Figure S5B provides additional ID-graphs created for genes that were used as target genes in the linear models, namely the *TMEM220*, *TGFBR3*, and *SHE* genes.

### 2.8. Epigenomic Regulatory Spatial Model

The expression of a gene is directly associated with the distances between its body and regulatory regions, and these distances differ in three-dimensional space as compared to the linear space [80]. Therefore, measurable and quantitative variations in spatial distance are subsequently responsible for changes in gene expression. Epigenetic processes, such as DNA methylation, impact the transcription machinery to influence gene expression [81]. Therefore, the spatial proximity of genes to some of the features identified as significant in the main MCFS-ID analysis, as discerning between breast cancer and normal samples, was verified at the level of 3D chromatin structure.

**Figure 7:**
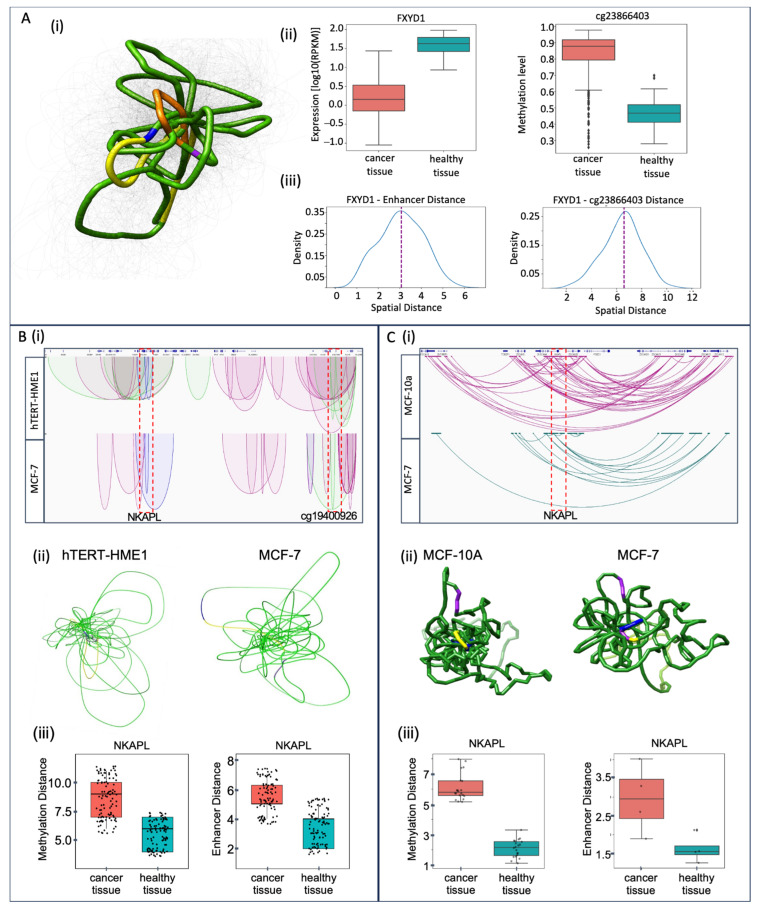
Spatial Regulatory Model of chromatin. (**A**) (**i**) The most representative chromatin 3D computational model from the ensemble of 100 spatial models generated by the 3D-GNOME method for the *FXYD1* gene with labeled promoter (blue), gene body (yellow) cg23866403 methylation loci (purple) and potential enhancer region (orange). (**ii**) The box plots show cancer and healthy samples *FXYD1* expression (**left**) and cg23866403 loci methylation levels (**right**). (**iii**) The spatial distance distribution between the FXYD1 gene promoter and its enhancer region (**left**) and the cg23866403 methylation loci (**right**). (**B**) (**i**) Cohesin-mediated chromatin interactions around the *NKAPL* gene in the integrative genomics viewer for hTERT-HME1 (healthy) and MCF-7 (cancer) cell lines. Green color annotates enhancer–promoter loops, blue color promoter–promoter loops. (**ii**) The representative chromatin 3D model based on Cohesin ChIA-PET data for the *NKAPL* gene. (**iii**) The spatial distances between promoter-methylation (**left**) and promoter-enhancer (**right**) for cancer and healthy cell lines. (**C**) (**i**) PCHi-C interactions around the *NKAPL* gene in the integrative genomics viewer for MCF-10A (healthy) and MCF-7 (cancer) samples. (**ii**) Chromatin 3D model of the *NKAPL* gene in MCF-10A (**left**) and MCF-7 (**right**) cell lines. (**iii**) The spatial Euclidean distances between the *NKAPL* gene body and DMS (**left**); the *NKAPL* gene body and the enhancer (**right**) both for MCF-7 and MCF-10A.

In the mass MCFS-ID analysis, several genes, whose expression was well-predicted by DNA methylation levels, were found. One of them was *FXYD1*, which was selected as an example to visualize the putative functional association between regulatory features and mRNA expression in 3D. (Figure 7A(i)). To achieve this, an ensemble of 100 models was built using the 3D-GNOME approach [82,83]. From this ensemble, the most representative spatial conformation was selected for visualization purposes in UCSF Chimera [84]. The gene promoter region and cg23866403 DNA methylation loci were observed to be in close proximity to the enhancer of the *FXYD1* gene in the normal sample, as compared to the longer distance between the regulatory elements in breast cancer, where *FXYD1* gene is down-regulated and cg23866403 loci are hyper-methylated, as shown in the box plots (Figure 7A(ii)). The spatial distance distribution between the enhancer–promoter and the methylation site-promoter was also calculated to see how these distances vary within the ensemble of 100 models (Figure 7A(iii)).

To thoroughly investigate the contrast between the 3D structure of cancer and normal cells, a model was constructed around the *NKAPL* gene, which contributes considerably to the classification of normal and tumor tissues [54,77] (also shown in the previous Section 2.7). To do this, two specific datasets (ChIA-PET and PCHi-C) were considered. The ChIA-PET dataset identifies 3D contacts (in this particular experiment, mediated by the RAD21 protein), which provides a chromosome-wide 3D view of the target gene and its spatial connectivity. The PCHi-C experiments provided a promoter-centric view of a target gene, with interactions between each gene promoter regions and other distal DNA segments, including regulatory elements.

Cohesin-mediated chromatin loops were explored by applying chromatin interaction analysis with the use of paired-end tag sequencing data (ChIA-PET) downloaded from Encode [85] for two cell lines (MCF-7 (cancer) and hTERT-HME1 (normal)) aligned to the hg38 reference genome. It was confirmed that the identified cohesin-mediated loops (significant interactions) connecting two distant genomic fragments (anchors) surrounding the *NKAPL* gene were stronger and higher in number in hTERT-HME1 (normal) than the MCF-7 (cancer). Moreover, the visual examination of the loops in the genome browser illustrates that loops anchored by distal enhancers were confirmed only for hTERT-HME1 (Figure 7B(i)). These enhancer-specific interactions in the hTERT-HME1 cell line work together with the promoter to control the expression of the *NKAPL* gene, and they are absent in the MCF-7 cell line (Figure 7B(i)). Additionally, 3D models from ChIA-PET were constructed using 3D-GNOME algorithm to examine and annotate the resulting structures (Figure 7B(ii)). We generated a spatial model of the *NKAPL* gene region for both cell lines by 3D-GNOME; these models suggest that chromatin in this regions is more condensed in the normal cell line compared to the cancer cell line, in which it is loosely packed in three-dimensional spaces (Figure 7B(ii)).

Next, the spatial distances in Euclidean space between (1) the *NKAPL* gene body and DNA methylation sites or (2) the *NKAPL* gene body and the enhancer were calculated for two cell lines, MCF-7 and hTERT-HME1 (Figure 7B(iii)). These distances were obtained by mapping the beads from the 3D model representing the gene body (*NKAPL*), the enhancer (*NKAPL*), and DMSs (Table S15, Figure S7), and by calculating them for respective pairs of beads. The hypothesis that the chromatin around this gene is more loosely packed in the cancer cell line than in the non-cancer one was confirmed by the box plots of the distance distributions (Figure 7B(iii) and Figure S7) for MCF-7 and hTERT-HME1 Cohesin ChIA-PET interaction sets.

To further investigate the differences between the 3D structure of this locus in cancer and normal cells, we examined promoter interactions with distal regulatory elements using PCHi-C datasets obtained from Javierre et al. [86] and Beesley et al. [87,88]. In PCHi-C, 23 interactions around the *NKAPL* gene in MCF-7 and 91 interactions in MCF-10A (normal tissue) were detected (Figure 7C(i)). In this case, 3D models were constructed using the Spring Model, which is based on the OpenMM molecular dynamics simulation engine based on a beads-on-string representation [89] (Figure 7C(ii)). Additionally, both 3D models are showing the loci with differential DNA methylation around the *NKAPL* gene, reflecting higher DNA methylation in cancer (Table S15, Figure S7). Again, the distributions of the Euclidean distance (calculated as previously for ChIA-PET data) confirmed the hypothesis that chromatin in this locus is more loosely packed in cancer than in normal cell lines (Figure 7C(iii)). The high concordance between the resulting box plot for PCHi-C and that of the ChIA-PET dataset confirms the reliability of the previous result.

## 3. Discussion

In this study, the MCFS-ID algorithm was applied to return a ranking of statistically significant molecular features distinguishing cancerous and normal tissue samples deposited in The Cancer Genome Atlas (TCGA) [https://www.cancer.gov/tcga] (accessed on 1 January 2020). Using this algorithm, we could select a small number of multi-omics features significantly different between cancer and normal samples, reducing the dimensionality of these datasets from 417k initial features to only 2.7k of the ranked features. Our further effort focused on verifying whether these significant features also had a substantive meaning in the context of cancerogenesis. It was shown that almost all (*n* = 590) mRNA significant genes returned by MCFS-ID reflected differential expression (Figure 1A) between cancer and normal samples. Nevertheless, MCFS-ID also explores the interactions; therefore, the statistical significance of individual features for the entire group of samples is not obvious. Meanwhile, a large part of the DMSs did not show differential methylation levels (Figure 2C). This suggests that mRNA-significant features may be standalone breast cancer predictors, while DNA methylation loci features must be considered in interaction with others to obtain highly predictive features (Table 1). This finding seems logical in the context of the regulatory functions of DNA methylation. The top *n* = 10 of 590 significant mRNA genes from MCFS-ID ranking were verified and confirmed (based on the literature) to have a meaningful impact on cancerogenesis, which implies that the choice of the implemented feature selection approach was meaningful. In the METABRIC cohort [90], the authors reported significant enrichment of immunologically related genes, which was also confirmed in our study, showing 79 of 590 mRNA genes being related to immunological processes. Over 75% of 590 mRNAs were down-regulated in cancer samples, which suggests that DNA hypomethylation in cancer leads to de-repression of gene expression, although such a high participation of down-expressed genes in cancer is quite surprising compared to the previously described patterns [91]. The majority of mRNAs that exhibited up-regulation in cancer samples contributed to cell cycle progression and mitosis. This finding is consistent with the characteristics of cancer cells, namely accelerated cell division and growth. However, such a generalization may lead to erroneous conclusions. We found that the group of up-regulated genes, namely *CCNB2*, *CCNB1*, *PLK1*, and *CDK1*, was associated with the Reactome pathway “Activation of NIMA Kinases NEK9, NEK6, NEK7” (Table S2). Up-regulation of *PLK1* in some of the breast cancer subtypes may inhibit tumor development by interfering with cytokinesis and mitosis [92,93] as well as one of the NIMA kinases *NEK9*, which is associated with tumor growth prevention, when upregulated [94]. Additionally, a group of genes from cluster 5, identified with the Natural Language Processing (NLP) clustering analysis (Table 2), showed a link with tumor suppressors, e.g., the *APC* gene that, through association with other proteins, prevents the uncontrolled growth of cells [95]. Concluding, depending on the biological context, up-regulated genes may result in activation as well as repression of processes contributing to cancer development.

As mentioned before, the majority of significant mRNAs (*n* = 590) were down-regulated in cancer cells. Pathway analysis showed that they can perturb the regulation of a wide range of Reactome metabolic pathways, including ethanol oxidation [96] and lipid metabolism [97,98], which are linked to breast cancer development, and also retinoid acid or neurotransmission, which are specifically connected with breast cancer treatment methods [99,100,101,102]. In addition to the results from the Reactome database, the NLP clustering approach unveiled a very interesting group of genes down-regulated in cancer (cluster 3, Table 2) associated with G protein-coupled receptors (GPCRs), which are cell surface receptors that detect ligands outside of the cell and initiate cellular response [103]. Moreover, in pathological states, GPCRs are over-expressed and activated in an aberrant way. This may imply certain aspects of cancer, including growth, invasion, migration, angiogenesis, and metastasis [104,105]. In breast cancer, multiple specific GPCRs were confirmed to participate in a plethora of autocrine and paracrine physiological effects or through activation of various ligands modulate cellular functions, which was associated with mRNA gene over-expression (revised in [106,107]). Interestingly, the genes from cluster 3 related to GPCRs were down-regulated, which is in contrast to known studies and that inconsistency should be tested in vivo. Another group of genes (cluster 2 Table 2) was described by keywords related to the ATP-binding cassette transporters (ABC transporters), some of which are involved in ion transport crucial for muscle contraction and cardiac processes. The majority of genes in this group were down-regulated in our cancer sample data. The literature provides evidence linking ABC transporters to single terms returned also by our NLP analysis such as ‘ions’, ‘transport’, ‘muscle’, ‘contraction’, and ‘cardiac’ with cancer biology [108]. For the ABC transporters family, both decreased and increased expression levels may be unfavorable for cancer patients, and some genes within this family are already molecular targets for anticancer drugs [109,110]. In this study, their levels were decreased in cancer samples. This reduction has been reported to contribute to more aggressive cancer forms, for example, as shown in *TMPRSS2-ERG*-negative prostate cancer [111]. For instance, reduced *ABCA9* expression in epithelial ovarian cancer has been linked to significantly shorter time to progression [112]. Interestingly, in patients with breast cancer, the reduced *ABCA8* expression lowers 5-year patients survival rate, is present in older patients (>60 age) as well as in the three breast cancer subtypes: ER-negative, PR-negative, HER-positive [113]. Among the keywords describing the remaining clusters of genes (Table 2), both general and very specific phrases were present. It seems that NLP based clustering analysis allows for efficient linking of even small gene groups with their related processes, which we find a big advantage. The obtained clustering results allow for a precise selection of genes having a general role from once having specific functions Further development of this NLP-based gene ontology analysis seems promising, especially as NLP is already widely and successfully used in other fields, e.g., supports disease diagnosis [114].

In breast cancer samples, DNA hyper-methylation in the regulatory regions of tumor suppressor genes and hypo-methylation of oncogenes has been shown [115]. In this study, MCFS-ID returned a ranking of 2006 DMSs that were significant predictors of breast cancer. These DMSs were located noticeably more often in CpG Islands (CpGI) and open seas, and less often in shores, than would be expected by the cytosine distribution in the entire Illumina panel. This result suggests that the genomic location of a cytosine influences its likelihood of being identified as a significant site, differentially methylated in breast cancer within our analytical framework. Overall, a small fraction of the returned significant DMSs was hypo-methylated in cancer samples (Figure 2C), while the majority was hyper-methylated. DMSs located in CpGIs were the most differentiated between normal and tumor samples in terms of methylation level (Figure 2B). The observed enrichment of DMSs within CpGI and their inverse correlation with mRNA expression, exemplified by the hypermethylation in the *NKAPL* promoter leading to its downregulation, highlights a precise regulatory mechanism. The above corresponds very well to the pattern of generally dominant DNA hyper-methylation in breast cancer and only local disorders of DNA hypo-methylation (revised in [116]). Furthermore, the distinct distribution of hyper- and hypo-methylated DMSs across various chromatin states—with hypermethylation prevailing in transcriptionally inactive regions and hypomethylation in active ones—provides a compelling epigenetic signature. This suggests that the interplay between specific methylation patterns and chromatin accessibility significantly contributes to the altered gene expression landscape characteristic of breast cancer, underscoring the potential of these specific methylation events as key drivers or indicators of tumorigenesis.

Moreover, we discovered putative functional associations between 59 DMS–mRNA pairs, out of which over 90% of these DMSs were located in distal genomic regulatory regions. DNA methylation changes in these DMSs may potentially affect the activity of enhancer-like regions, influencing the target-gene expression. Out of 34 genes under such enhancer-like regulatory effect, only two (*KIFC1* and *HN1L*) were over-expressed in cancer, both with significant negative correlation with DNA methylation. Up-regulation of *KIFC1* expression is well known for breast cancer [117], and the protein has been suggested as a chemotherapy target [118], while over-expression of *HN1L* is related to tumor invasion in breast cancer [119]. Next, based on the discovered association among the DNA methylation sites within the promoter of *NKAPL* gene and TFBS of NRF1 TF shown in this study, we are confident about the presence of putative functional dependency of these molecular elements. The proposed regulatory model suggests that hypermethylation at five cytosine loci within the *NKAPL* promoter may obstruct NRF1 binding, either by diminishing its affinity [120] or through MBD protein binding, resulting in its down-expression in tumor samples. MBD proteins can bind to specifically methylated cytosines, preventing other factors from binding and simultaneously may co-form complexes (for example, MBD2 and MBD3 in the NuRD complex), which may lead to gene silencing [121]. Moreover, based on the results from the extensive use of MCFS-ID (Table S9), it was possible to select 23 cytosines significantly associated with *NKAPL* expression (r = 0.81). Among all of them, only one cytosine (cg18675097) was located within the *NKAPL* promoter, and all the others were located (up/down-stream) at least 1,369,587 bp from *NKAPL* TSS, suggesting their presence in distal regulatory regions of *NKAPL*. By using this approach, we additionally selected 66 mRNA genes whose expressions were well-predicted by DNA methylation, and 39 of them were well-predicted by miRNA expressions as well (see Section 2.5).

To review the significant set of *n* = 105 miRNA returned by MCFS-ID, the analysis of associations between miRNA to mRNA was conducted resulting in the list of proteins targeted by drugs used for breast cancer treatment. For example, palbociclib (DB09073), a drug for treating metastatic breast cancer which targets proteins encoded by genes such as *CCNA2*, *CCND1*, *CDC25A*, *CDK1*, *CHEK1*, *ESR1*, *KRAS*, and *PLK1* has a DScore of 0.99, while the GScore of the genes is 0.85. At the same time, other drugs with high DScore, used in breast cancer treatment, e.g., ribociclib [65], abemaciclib [66], tamoxifen [122], etc., were found to be linked to miRNA reported as significant in this study (Figure S3C).

Methylated/unmethylated nucleotides within the TFBS may disturb TF binding to DNA sequence. This may change TF binding affinity, and shift the factor binding site, resulting in alternative protein complex formation, binding prevention or other TFs binding to such locus [123,124]. At the same time, it is not clear what the order of these events is, what initiates the process, and what the results are [24]. In this study, we identified many more TF motifs overlapping hyper-methylated DMSs than hypo-methylated, which reflects a much higher frequency of hyper-methylated cytosines among the 2006 DMSs. Moreover, almost all motifs, except EPAS1, containing hypo-methylated cytosines were identical to the TF motifs containing hyper-methylated cytosines. There are reports indicating that EPAS1 may support proliferation and migration and increase tumor cell invasiveness [125,126]. Genes encoding TFs that bind to motifs identified in sequences containing hyper-methylated cytosines (Figure 5D) belonged to, among others, cell cycle-signaling pathways, transcription misregulation in cancer, the TGF-β pathway, cellular senescence, and several cancer types, including breast cancer q-value = 0.0000234 (Figure 5D and Table S10).

In the second MCFS-ID experiment, the association between DMSs and the expression of genes encoding TFs, whose motifs overlapped these DMSs, was exposed. There were seven genes encoding TFs: *MXI1*, *EPAS1*, *PLAGL1*, *E2F1*, *NR0B1*, *BHLHE41*, and *ARNT2*. *PLAGL1* has been reported as a possible epigenetically regulated tumor suppressor gene [127], and *NR0B1* (also known as *DAX-1*) has been repeatedly indicated as a potential target for anticancer therapy in patients with breast cancer [128,129]. Likewise, there is evidence that low expression of *BHLHE41*, which was also observed in the results of this study, promotes breast cancer tumor invasion [130]. The other four (*EPAS1*, *MXI1*, *ARNT2*, and *E2F1*) relate to the signaling pathways involved in the processes of tumor formation and development [131].

Using the epigenetic variables that functionally interact with each other, here DMSs and TFs, together with the MCFS-ID and linear regression models, allowed for the identification of mRNA target genes under probable epigenetic regulation (see Figure 6A, Table S12). Among nine target genes, four were confirmed to have linear models with high goodness of fit: *TMEM220*, *NKAPL*, *SHE*, and *TGFBR3*. These genes were down-regulated in the tumor samples and seemed to be tumor suppressors whose activity could be regulated by DNA methylation located within TFBS of specific TFs.

Hyper-methylated DMSs may reduce these four TFs’ binding affinity and change gene expression. Moreover, these DMSs are located in heterochromatin regions that are known to contribute to gene silencing. *NKAPL* (NKAP-like) is a cell-specific transcriptional suppressor in Notch signaling [132], and its reduced expression in cancer has been indicated by several articles, just as the relationship between DMS hyper-methylation and the demonstrated change in *NKAPL* expression [77,133]. Transforming growth factor beta receptor III encoded by *TGFBR3* binds inhibin and can mediate functional antagonism of activin signaling [134]. Decreased expression of *TGFBR3* (former *ETDL1*) causes decreased TβRIII expression in tumor tissues, resulting in tumor progression due to increased invasiveness, angiogenesis, and a chance of metastasis [135,136]. Next, the SH2 (SHE) domain-containing adapter protein E possesses the Src homology 2 (SH2) domain identified in the oncoproteins Src and Fps. It functions as a regulatory module of intracellular signaling cascades by interacting with phosphotyrosine-containing target peptides [137]. The Transmembrane protein 220 (*TMEM220*) is involved in the FOXO and PI3K-Akt pathways [138] and promotes regeneration [139]. The down-expression of *TMEM220* and *SHE* genes (also in connection with hyper-methylation) has been repeatedly indicated as a significant factor important in the formation and development of cancer but not necessarily breast cancer (Refs. [44,138,140] and Supplement Table S1 in [141]). Our results are, therefore, the first to indicate the impact of these two genes in breast cancer development.

To extend the analysis of functional importance of the four genes, the gene–gene interactions were used to build the network of 17 new genes connected to the initial four (Figure 6B). The functional investigation of genes with the highest number of connections (*MYC*, *NOG*, *TGFB1*, *VIRMA*, *SRC*, and *AR*) in the network showed an overrepresentation of signaling pathways related to cancer processes, associated mainly with cell cycle disorders, namely proliferation, growth, differentiation, migration or apoptosis, as well as patients survival. The returned KEGG pathways were consistent with the previously discussed gene’s biological functions. For example, the hyperactivation of the Mitogen Activated Protein Kinase (MAPK) pathway is frequently observed in many cancers, including breast cancer. It is an oncogenic pathway, and at the same time, it is crucial for the signal transduction of the ErbB protein family [142]. Some proteins from the ErbB family are oncogenes associated with proliferation and apoptosis. They are also related to cancer treatment resistance in some breast cancer subtypes [143]. At the same time, the MAPK is associated with PI3K-AKT-mTOR, i.e., the pathway that is directly related to *TGFB1* and *MYC* and indirectly with *TMEM220* and *AR*. PI3K-AKT-mTOR is associated with the processes of oncogenesis and breast cancer development, and many inhibitors of this pathway are currently in clinical trials [144,145]. Another overrepresented pathway was Hippo, which is linked with proliferation, migration and apoptosis [146], and metastasis changes [147]. The Hippo pathway is well-known for having an impact on the transforming growth factor beta (TGF-β)-signaling pathway through which they may control tumor development [148,149]. Additionally, components of the TGF-β pathway play a significant role in the proliferation, cell growth, and differentiation of cells, but also affect the immune system, enabling the repair or development of ongoing processes that were shown to negatively affect the patient’s condition [148,150,151]. Members of the TGF-β protein family play an essential role in apoptosis and migration, the regulation of which can have a vast impact on breast tumor development, especially at its later stage [148,151,152]. Additionally, one of the miRNA genes (hsa-miR-211), which was pointed out as significant in the presented regulatory network (Figure 6A, Table S5), is known to participate in the TGF-β pathway [153]. The hsa-miR-211 is involved in the regulation of proliferation, migration, invasion, apoptosis, and drug resistance [154], and we discovered its association with the *SHE* gene. Alterations in the expression level of hsa-miR-211 have been repeatedly reported in the context of various cancer types, but the direction of its expression level changes depending on the type of cancer. *SHE* was confirmed as an oncogene and/or tumor suppressor, depending on cancer type [155]. In breast cancer, change in *SHE* expression, no matter if decreased or increased, results in metastasis and poor prognosis [154]. We are convinced that the analysis of hsa-miR-211 with the *SHE*, whose role is little known, should become the subject of detailed studies. Additionally, one of the terms related to the obtained gene–gene network was “chemical carcinogenesis”, which is connected to many environmental and chemical factors, having a strong impact on the oncogenic processes including DNA methylation. Therefore, there are multiple indirect confirmations that the created network demonstrates an interplay among the detected epigenetic disorders, which, in turn, leads to subsequent changes affecting target gene expression and disease development.

From the massive MCFS-ID computational approach, we discovered an association between cg23866403 loci and *FXYD1;* to verify this functional putative association, we built a chromatin spatial model using the 3D-GNOME approach. Based on the obtained results (Figure 7), we hypothesize that, in the normal tissue, the lower level of cg23866403 loci methylation in the *FXYD1* gene promoter results in a shortened spatial distance to the gene enhancer, and, as a result, *FXYD1* increased expression compared to the cancer tissue. The significant impact of DNA methylation on the chromatin structure in cancer is well-studied [156], showing the appearance of changes, for example, due to CTCF binding disruption [157]. Moreover, the length of the DNA loops may change depending on the cohesion’s presence in gene expression regulatory machinery as well as the presence of CTCF in one or both anchors of a loop [158]. The relationship between distance change and gene expression change is very poorly understood; the only example we found was in *Drosophila* [159]. Based on our results, one could suggest that the change in DNA methylation affected protein binding; consequently, it changed the length of the loop. Through the results presented in this study, the *NKAPL* gene was found to appear in multiple contexts, making it an interesting target, especially in its transcription relation due to altered DNA methylation. Therefore, we built spatial models for it using two different experimental chromosome conformation capture protocols, namely ChIA-PET and PCHi-C (Figure 7). The first model allowed us to observe the change in loop length and discover that cohesin-mediated loops surrounding the *NKAPL* gene were longer in the normal cell line (hTERT-HME1) than in the cancerous (MCF-7) and also that loops anchored by enhancers are present only in the normal cell line. Thanks to the second model, it was noticed that in the normal cell lines (MCF-10A) around the *NKAPL* gene, several times more interactions can be identified compared to the cancer cell line (MCF-7), with a similarly higher level of DNA methylation in cancer. Based on the spatial models obtained by both methods, we hypothesize that the reduced level of methylation in normal cells results in the formation of a much larger number of stable interactions, which translates into a more condensed chromatin region. However, this increased level of chromatin condensation in the normal cell line cannot be interpreted as closed chromatin, preventing transcription and expression regulation processes. It is tighter because of the higher number of established connections. In contrast to the cancer cell line, where fewer connections result in looser chromatin structure, putatively unstable, and, accordingly, exposed to unexpected transcriptional changes that may result in the development of potentially oncogenic changes. Chromatin organization has a significant impact on organism functioning [160], and its disruption may support pathogenic processes, e.g., through chromosomal instability, which intensifies deregulation of gene expression [161]. Changes in DNA looping can cause errors in gene regulation during both cancer initiation and development [40,162]. Indeed, alterations such as the absence of specific loops or shift in chromatin interaction frequency have been directly linked to carcinogenesis in various cell lines [85]. Moreover, a more loosely packed chromatin state may reduce nuclear stiffness and increase chromatin mobility [163,164]. This enhanced mobility can lead to chromosomal translocations and changes in the transcriptional landscape, which may contribute to oncogenic events [161,165]. This mechanism may explain why we observed more loosely packed chromatin in the cancer cell line specifically related to the *NKAPL* gene promoter in our study. Beyond these structural changes, cohesin-mediated chromatin structures are known as regulators of Epithelial–Mesenchymal Transition (EMT)-related genes, whose altered expression influences cancer progression, including breast cancer [166]. EMT itself may also be influenced by disturbances in the TGF-β-signaling pathway, which may suggest the multilayered nature of cohesin-mediated chromatin structures disorder [166,167]. Finally, it must be noticed that we used TCGA tissue samples, and they represent various cell heterogeneity. This may influence the study, because some of the observed values might be averaged and not confirmed as significant. At the same time, we are certain that the altered transcriptomic and epigenetic signals presented in this work are of great value; when studied further, at the level of specific cell populations, they will bring detailed insight into gene expression regulation during breast cancer development.

## 4. Materials and Methods

### 4.1. Data Collection

**Figure 8:**
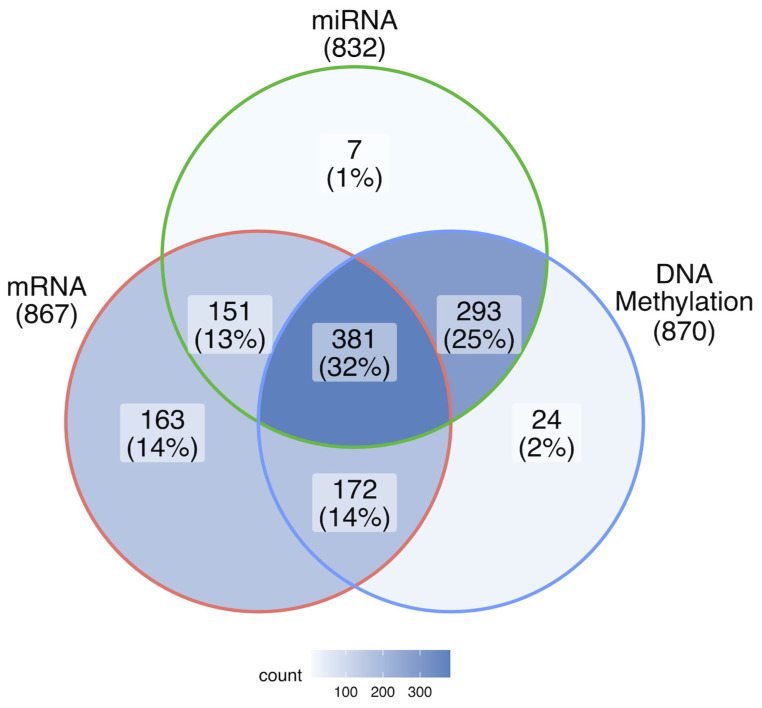
Number of samples with the complete data for a given dataset type. The overlap of 381 samples that contain the complete data for all three dataset types was used in the feature selection procedure.

This study is based on breast cancer data obtained from The Cancer Genome Atlas (TCGA) including mRNA, miRNA expression, and DNA methylation levels [https://www.cancer.gov/tcga] (accessed on 1 January 2020). The data was filtered as follows: (1) all attributes having zero variance across samples were removed; (2) only female samples were included in the study. The final dataset consisted of 1191 samples taken from 1068 female patients (123 patients donated both normal and cancerous tissues). Out of the 1191 samples, only 381 were complete among mRNA, DNA methylation, and miRNA data. They consisted of 328 cancerous and 53 normal samples (Table 4 and Figure 8). The remaining set of samples (incomplete among all datasets) were used as testing sets in separated classification experiments described in Section 4.2 and Section 2.1.

### 4.2. Detection of Significant Features Using MCFS-ID Algorithm

Our analysis utilized the Monte Carlo Feature Selection and Interdependencies Discovery (MCFS-ID). This algorithm allows the user to perform a supervised feature selection introduced in [168]. MCFS-ID generates a ranking of features based on their potential to distinguish records between classes, e.g., cancerous vs. normal. It also enables the prediction of continuous values and allows the user to discover possible interdependencies between features.

The algorithm builds thousands of decision (or regression) trees on randomly selected subsets of data samples and attributes. The relative importance (RI) score for each feature is calculated based on all decision trees and nodes built on that feature: the number of samples split by the node, the information gain of the node, and the predictive quality of the trees. The RI score is used to build the ranking of all input features. The ranking signifies which attributes are best to use in classification or regression tasks. Additionally, the algorithm provides an RI cutoff that assures that attributes that exceed it are better for predictions than attributes with random values, which might distinguish classes by pure chance. The upper part of the feature-ranking cut-off by the RI value constitutes the significant features set.

The Interdependency Discovery function of the MCFS-ID algorithm allows the user to find links between features that amplify each other in the classification task. Decision or regression trees used for calculation of RI scores are also used to generate feature interdependency scores. In this case, the score for the pair of features (parent/child nodes in the tree) is generated using information gain of the child node multiplied by its associated number of samples expressed as a fraction of samples in the parent node.

The resulting scores indicate which features amplify the prediction powers of other features. The result of this algorithm has the form of a directed graph where features are visualized as nodes and the thickness of edges symbolizes the strength of this amplification. For better clarity and to avoid false positives, MCFS-ID allows us to cut off edges that are weaker than those that might be caused by random patterns occurring in the data. Interdependency graphs were generated using the ‘build.idgraph’ function from the ‘rmcfs’ package. For more details of the MCFS-ID, see [7].

**Figure 9:**
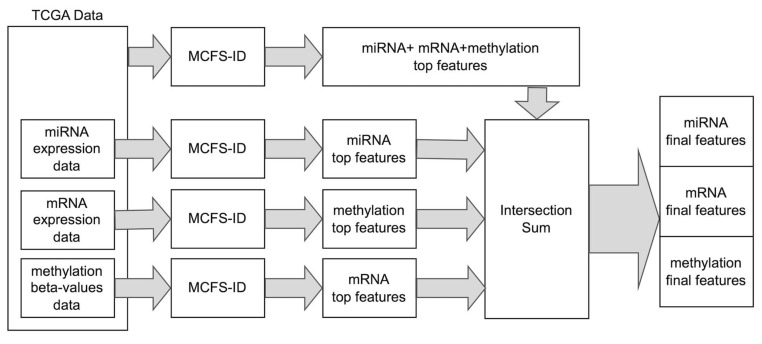
Selection of features identified as significant in cancer prediction (the main MCFS-ID experiment).

To establish a final list of significant features for each data type, four runs of the MCFS-ID algorithm were performed (Figure 9).

First of all, a selection of features was performed on the full dataset consisting of mRNA expression, DNA methylation, and miRNA expression in 381 samples. This approach allowed for capturing the relationships between attributes from different types of datasets. Next, a feature selection was performed on the same dataset but separately for each data type (Figure 9), where the sample size differed depending on a data type (Table 4, Figure 8). In all of these four runs, the features were selected by their ability to differentiate between normal and cancerous samples. Running MCFS-ID on the data of all types allowed us to find features significant in conjunction with features of other types. Attributes derived from this run are to be called significant, along with all categories. Separate runs for each data type capture the less (but still) important features that would be under the relevance threshold of the MCFS-ID algorithm in case they were part of a larger set of features. The whole procedure (Figure 9) will be referred to as the main MCFS-ID experiment later in the paper, and the features selected by the procedure as the significant set of mRNA/miRNA genes or DNA methylation features. The analysis was conducted using version 1.3.1 of the ‘rmcfs’ package from the CRAN repository in R 3.6.3. The default parameters were used except splitSetSize = 200; mode = 2; cutoffPermutations = 20.

Additionally, to validate the quality of the selected important features, two different classification models (SVM (support vector machine) and RF (random forest)) were trained on the 381 complete samples and tested later on the test samples that were not used in the feature selection phase. For each type of data, there are over 400 unique test-ready samples that do not contain values for all categories; these were used to obtain weighted accuracy of classification (wAcc) for each category separately.

### 4.3. Descriptive Analysis of Significant mRNA Genes

To annotate genes as down- or over-expressed in the cancer samples, the log2 fold change was calculated for each of the significant genes (returned by MCFS-ID). The distribution of gene expression was verified with the Shapiro–Wilk test, and, depending on the obtained results, the Wilcoxon test was applied. Next, functional annotation of significant genes was performed using the Enrichr web server [169], followed by the Benjamini–Hochberg correction for multiple testing. Initially, the q-value was set to 0.05, but because of a huge number of significant terms returned, in one (over-expressed mRNA) analysis, the threshold was set to 0.001. Moreover, based on the set of significant genes descriptions, gathered from various molecular databases provided by BioMart (such as NCBI, Gene Ontology, KEGG, Reactome, WikiPathways, Biocarta), Natural Language Processing (NLP) methods were applied to group these genes into functionally/descriptively similar clusters and retrieve sets of key words to describe each of the returned cluster. To perform this operation, each gene was represented as the text document (combined from all available descriptions), and, later, used to calculate TF-IDF (term frequency–inverse document frequency) as a bag of words/terms. When each gene is represented by a TF-IDF vector, it is possible to calculate cosine similarity between the documents and a hierarchical clustering model can be built based on these similarities. Finally, discovering a set of unique keywords that characterize each cluster provides a good functional biological overview of the input genes and groups that should be studied more closely.

### 4.4. Descriptive Analysis of Significant DNA Methylation Sites

DNA methylation sites selected by the MCFS-ID (hereafter differentially methylated sites, DMSs) were mapped to the specific genomic regions. Original DMSs positions were converted from hg19 to hg38 using LiftOver [170]. Intersections of genomic regions, i.e., CpG islands (CpGIs) or promoters with DMSs were performed using bedtools (v2.29.2) [171]. The locations of CpG islands (CpGI) were taken from the UCSC database represented in hg38 genome assembly (https://hgdownload.cse.ucsc.edu/goldenpath/hg38/database/cpgIslandExt.txt.gz, accessed on 1 November 2019). The shores were defined as the regions flanking the CpGI by 2000 bp up- and down-stream, the shelves as the regions flanking the shores by +/−2000 bp, and the open seas as the regions between the shelves. Next, to prepare the background distribution of cytosines across these four region types, all cytosines included in the Human Methylation 450K BeadChip (Illumina 450K) were mapped to CpGIs, shores, shelves, and open-sea regions. The sites covered by the Illumina 450K represented the background distribution of cytosines across the named genomic regions and allowed to verify (chi-squared test) whether DMSs show any specific distribution. Afterwards, the distribution of *β*-values of cancer vs. normal samples was compared separately for CpGIs, shores, shelves, and open-sea regions using the Wilcoxon test. *β*-values represented the ratio of the intensity of the methylated bead type to the combined intensity of a locus and obtained values between 0 and 1 [172]. Finally, we tested whether the distributions of hypo-, medium-, and hyper-methylated cytosines in cancer samples differed across the four region types (chi-squared test). Bonferroni correction was applied for multiple testing corrections for the aforementioned analysis.

To annotate DMSs to promoters or gene bodies, the gene positions from Ensembl (https://www.ensembl.org/Homo_sapiens/Info/Index, accessed on 1 February 2020) were taken, and their promoters were set to +/−2000 bp around the TSS. DMSs assigned to the promoter or the gene body established a pair: a DMS and its target gene. DMSs within intergenic regions were paired with their target genes using the bedtools closest function. Assignment of hyper-, hypo-, and medium-methylated *β*-values was performed on the basis of the log2 fold change (log_2_FC) between sample types (cancer vs. normal). Sites with log_2_FC ≤ −1 were labeled as hyper-methylated in cancer and log_2_FC ≥ 1 as hypo-methylated DMSs in cancer; the remaining sites were labeled as medium-methylated. To evaluate the significance of the observed log_2_FC, first the Shapiro–Wilk test was used to verify whether the data was normally distributed, and, based on the test results, a nonparametric Wilcoxon test with FDR correction was chosen to verify the null hypothesis that there was no difference in distribution of *β*-values for DMSs between cancer and normal samples.

To detect putative regulatory regions, the association between gene expression and the *β*-value of DMSs located 1 Mb upstream/downstream from that gene, TSS was verified by calculation of the Spearman correlation with FDR correction, defining significant correlations when Spearman’s |rho| ≥ 0.6 and FDR ≤ 0.05. Moreover, to assign DMSs to specific chromatin states, we used chromatin state annotations for the MCF-7 breast cancer cell line (GSE57498). The data were converted from the original annotation Human GRCh37/hg19 to GRCh38/hg38 annotation using LiftOver [170]. Positions indicating acetylation of the lysine 27 of the histone H3 protein (H3K27ac) for the MCF-7 cell line were taken from the ENCODE database (https://www.encodeproject.org/files/ENCFF621API/, accessed on 1 October 2020). The sites included in the Illumina 450K panel were intersected with the ranges of chromatin states to achieve the general distribution of cytosines across chromatin states. Next, from the significant 2006 DMSs, hyper- and hypo-methylated DMSs were selected and independently intersected with chromatin states to unveil their distribution across chromatin states. To verify whether the obtained distributions were specific, 2006 random loci were drawn 1000 times from the Illumina 450K panel. Each time, a set of those drawn loci was intersected with the chromatin state ranges to compute the percentage of loci assigned to specific chromatin state. Next, the logarithm of fold change between percentage of hyper-methylated DMSs and mean percentage of randomly drawn loci for each chromatin state was computed. The same procedure was applied to hypo-methylated sites, generating empirical *p*-values of significance for these overlaps.

Next, to evaluate the impact of DMSs on the patients’ survival, a multivariate log rank test (‘lifelines.statistics.multivariate_logrank_test’ function in Python 3.8) was used. The samples were split into high and low methylated groups, defined by a median of methylation level. Additionally, to confirm that the number of DMSs, discovered to have a significant impact on survival, differs significantly from the number of such sites selected randomly, a bootstrapping technique (sampling 100 times) was applied. After each sampling of 2006 random sites from all sites present in the Illumina 450K panel, their impact on survival was tested, and the number of sites with *p*-value below 0.05 was noticed. Next, we counted how many times the number of significant sites (having impact on survival) found in a random set was greater than the number of significant sites (having impact on survival) obtained for 2006 DMSs.

### 4.5. Descriptive Analysis of Significant miRNA Genes

To characterize the set of significant miRNAs, returned by the MCFS-ID, we verified their presence in the miR + Pathway database [173], which contains information about mRNA–miRNA connections and 150 KEGG pathways linked with mRNAs. The mRNAs linked with the searched miRNAs were intersected with 590 mRNAs returned in the MCFS-ID experiment. Biological functions of the resulting mRNAs were verified on NLP cluster keywords (described in Section 4.3).

Next, to study the suppressive impact of breast cancer on the expression of significant miRNAs (those returned in the main MCFS-ID experiment) and miRNAs’ regulatory role in mRNA gene expression, the selection of down-expressed miRNAs’ genes in cancer was performed based on log_2_FC (Figure S3A). The genes were defined to be down-expressed in cancer if log_2_FC ≤ −0.5 (Figure S3B). Next, for the selected miRNAs down-expressed in cancer, their mRNA target genes were assigned using the MicroRNA Target Prediction Database [174], and Spearman correlation was calculated for the obtained miRNA–mRNA pairs. The correlations where *rho* ≤ −0.2, and adjusted *p*-values ≤ 0.05 were considered in further analysis. Next, for the mRNA genes that significantly correlated with miRNAs using the STRING database [175], the protein–protein interaction identification was performed, and based on the number of interactions among proteins, the top 50 proteins were selected (with the highest number of interactions). Next, using KEGG pathways and gene ontology biological processes (GO BP), the enrichment analysis of those top 50 proteins was performed. Additionally, to discover putative drugs associated with down-expressed miRNAs, the Enrichr Database was searched with the same set of proteins.

### 4.6. Detection of Significant miRNA and Methylations in the Context of Predicting mRNA Expression Levels

**Figure 10:**
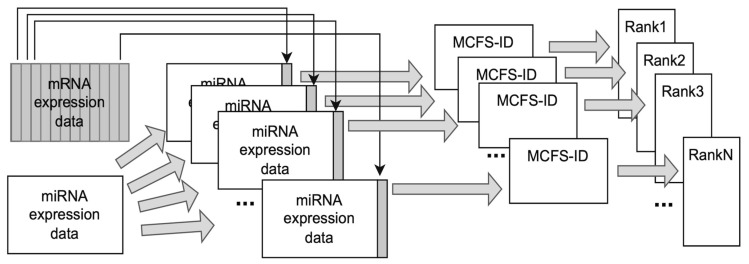
Selection of miRNA genes significant in prediction of mRNA gene expression levels.

To discover more complex interactions between the top 590 most significant mRNA genes (obtained from the main MCFS-ID experiment described in Section 4.2) and two other types of molecular data (DNA methylation and miRNA expression), two additional sets of MCFS-ID experiments were performed (Figure 10). Both sets of experiments were based on the same idea of running the MCFS-ID algorithm on all top significant 590 mRNA features obtained from the main MCFS-ID runs. Each of those mRNA features was used as a target variable, and miRNAs or DNA methylation were used as predictor features. Finally, two different feature rankings were obtained. In the case of DNA methylation, for each run on a different target mRNA gene, methylation loci within the chromosome of the gene were explored. This limitation heavily reduced the calculation time—the number of all DNA methylation sites is almost 400k and it is biologically justified to focus on such relations within one chromosome [176]. In both sets of experiments, the final cross validation of the result was based on a regression tree modeling and the calculation of Pearson correlation between the predicted value and the observed mRNA gene expression level.

For each significant mRNA (selected by the main experiment described in Section 4.2) treated as a target variable, a separate MCFS-ID experiment was performed. The same procedure was used for DNA methylation as predictors instead of miRNA expression levels.

### 4.7. Descriptive Analysis of Associations Between DMS and TFs

To predict transcription factors (TFs) whose binding affinity to DNA sequence may be changed due to differential DNA methylation, the sequences surrounding each DMS (+/−20 bp) were generated using the bedtools getfasta function (v2.29.2) [171]. To identify TF motifs, the HOCOMOCO database of position weight matrices (PWMs) [177] with the PWMEnrich tool [178] were used with the following settings: (i) sequences background build based on randomly selected fasta sequences and (ii) motif significance cutoff *p*-value ≤ 0.001. Next, to detect the exact Transcription Factor Binding Site (TFBS) positions of the motifs that passed the threshold, the online FIMO tool [179] from the MEME Suite 5.0.5 was applied with a significance threshold of *p* ≤ 0.0001. The returned TFBS were intersected with DMS to keep only these TFBS, within which any DMS was confirmed, to ensure that differential DNA methylation may affect binding affinity. To group TF motifs, returned by PWMEnrich, based on the heterogeneity of their PWMs, the STAMP tool [180] was used with Pearson Correlation Coefficient as a measure of distance. Sequence alignment was performed using an ungapped Smith–Waterman algorithm with the iterative refinement multiple alignment strategy. Visualization of the clustering results was performed with UPGMA [180]. Furthermore, functional annotation of genes encoding TFs was performed using the Enrichr web server [169] with the Benjamini–Hochberg correction for multiple testing and a significance threshold q-value of ≤0.05.

### 4.8. Building Models of Regulatory Networks

To investigate whether the putative associations between TFs and DMSs within the binding sites of these TFs had similar patterns in cancer vs. normal samples, the additional analysis using the MCFS-ID algorithm was performed. The input decision table consisted of 484 patients for whom both mRNA and DNA methylation datasets were available. Features, namely TFs’ coding genes and DMS within DNA sequences of motifs detected for those TFs, were analyzed. The returned significant features, together with cancer/normal tissue type, were used as explanatory variables to train a set of linear models to predict mRNA expression of a target gene. Each time, a different mRNA gene (target gene) whose promoter overlapped with a set of explanatory variables (TFs and DMS) was used as a dependent variable to build a single linear model. Finally, for the best-fitted models (adjusted *p* ≤ 0.05, R^2^ > 0.5), the feasible biological relationships between DMS, TFs, and their target genes were visualized. These target genes were also used to discover direct and the closest indirect associations between them, as well as other genes by the systematic literature review and interaction graphs obtained through the Pathway Commons online tool [181]. Based on the associations found, a final graph of connections between identified target genes and other genes was created and visualized.

### 4.9. The Visualization of Chromatin 3D Structure of Selected Loci

In order to visualize the putative functional association between genes, DNA methylations significant in breast cancer, and enhancer regions, the chromatin structures of the *FXYD1* and *NKAPL* loci were generated using 3D-GNOME [182] and Spring Model (SM) [89] polymer simulation methods.

3D-GNOME is a chromatin 3D structure modeling method that uses a multiscale bead-on-a-string approach and a Monte Carlo simulated annealing algorithm [182]. It models chromatin interactions mediated by specific proteins based on high-frequency PET (multiple paired end tags mapped on two genomic loci) cluster interactions and singletons (single paired end tag). The algorithm uses a tree structure to manage the relationships between different levels of genomic organization (chromosomes, segments containing a topological domain, and chromatin interaction anchors), simulating their spatial positions independently by minimizing an energy function based on high-frequency chromatin PET cluster interactions and energy terms. Next, sub-anchor beads are added between neighboring anchor beads to model chromatin loops, and their positions are again simulated by minimizing energy. Finally, the algorithm refines the loop shape using a singleton interaction heatmap and motif orientation. The 3D-GNOME models were generated based on cohesin mediated Chromatin Interaction Analysis by Paired-End Tag sequencing (ChIA-PET) data for the hTERT-HME1 (normal) and MCF-7 (cancer) cell lines (ENCODE Accession ID: ENCSR991JXX—hTERT-HME1, ENCSR255XYX—MCF-7) [85].

The Spring Model represents polymers as a collection of points in three-dimensional space using the beads-on-chain approach. In the resolution chosen by the user, each bead represents a segment of DNA of the same length. In this study, chromatin models with a resolution of 1 kbp were constructed, where each bead represented 1000 base pairs. If there was a spatial interaction between beads in the polymer model, harmonic bonds were used to connect pairs of interacting beads by springs. The spring-based pairwise forces were subjected to energy minimization in a SM polymer simulation. In order to establish the final 3D structure of the polymer fiber with the set of experimentally identified contacts, the SM simulation undertook the global energy minimization-given data-driven forces represented by the springs and polymer chain parameters (such as stiffness). The initial conformation of the polymer was given as a circular 3D structure of polymer fiber. Three-dimensional models generated by the Spring Model approach were built using Promoter Capture Hi-C (PCHi-C) [87] data for MCF-10A (healthy) and MCF-7 (cancer) breast tissues to achieve a promoter centric view.

## 5. Conclusions

This study successfully extracted a very rich set of multi-omic features, including mRNA expression, miRNA expression, and DNA methylation, enabling accurate classification of breast cancer vs. normal samples. The top-ranked features included many established breast cancer-related markers, validating our analytical approach. Simultaneously, we identified novel features, and we are certain that some of them represent potential novel targets for functional research in the context of breast cancer development and drug studies. Among the previously undescribed features, there were mRNAs (*NKAPL*, *PITX1*, and *TMEM220*) for which we proposed potential regulatory mechanisms based on epigenetic interactions. Furthermore, 3D chromatin structure models revealed a less condensed chromatin structure in cancer, suggesting significant regulatory dependencies. The presented bioinformatic approach, which effectively reduces data dimensionality, provides a robust framework for selecting key features for subsequent experimental validation.

## 6. Limitations of the Study

Despite identifying numerous statistically significant molecular features and their complex interdependencies related to breast cancer, our study has certain limitations that warrant discussion. Firstly, while the TCGA dataset is extensive and widely utilized, it is primarily based on bulk tumor tissue analysis. This approach inherently averages signals from heterogeneous cell populations, including tumor cells, stromal cells, and immune infiltrates. Consequently, our findings may not fully capture the nuanced, cell-type-specific molecular alterations that drive cancer, nor do they reflect the single-cell resolution required for a complete understanding of tumor heterogeneity and evolution. Secondly, although MCFS-ID efficiently identifies features with high predictive power, the biological relevance and causal relationships for many of them remain putative. While we provide extensive literature support and biological pathway analyses, direct experimental validation (e.g., in vitro or in vivo functional assays) for gene expression, methylation, or miRNA modulation was beyond the scope of this computational study. Such validation would be crucial to confirm their functional roles as biomarkers or therapeutic targets. Furthermore, the cross-sectional nature of the TCGA data limits our ability to infer temporal causality or track dynamic changes in multi-omics profiles over the course of disease progression or treatment. Lastly, while we explored 3D chromatin structures for specific examples like *NKAPL*, these analyses relied on established cell line data, which may not perfectly recapitulate the complex in vivo environment of primary human tumors. This limits the direct translatability of certain 3D chromatin findings to heterogeneous patient populations. Future studies integrating single-cell multi-omics data, experimental functional validation, and longitudinal patient cohorts will be essential to overcome these limitations and further advance the understanding and clinical application of these findings.

## Figures and Tables

**Table 1:** Significant features number returned by the MCFS-ID rankings together with RF and SVM classification results.

	Joined-Set	Individual-Set	Intersection	Sum	RF wAcc	SVM wAcc
DNA methylation	1504	1987	1485	2006	0.9068	0.9398
mRNA	432	588	430	590	0.9347	0.9347
miRNA	6	105	6	105	0.9848	0.9370

**Table 2:** The result of NLP clustering of the significant mRNA genes.

Cluster ID	Cluster Size	Top Words Associated with Genes in Cluster	Keywords Interpretation	Mean Log Fold Change for Verification Set	Direction of Change in Expression
1	393	regulation, process, metabolism, negative, negative_regulation, response, positive, positive regulation, gene, metabolic	regulation and metabolic processes	1.5894	over-expressed: 78 down-expressed: 313
2	38	transport, ion, transmembrane, transmembrane_transport, calcium, abc, muscle, cardiac, ion_transmembrane, contraction	ion trans- membrane transport	2.5636	over-expressed: 4 down-expressed: 34
3	18	receptor, g, coupled, g_protein, protein_coupled, gpcr, coupled_receptor, receptors, protein, ligand	receptor proteins	2.8793	over-expressed: 0 down-expressed: 18
4	24	transcription, polymerase, rna_polymerase, rna, polymerase_ii, ii, regulation_transcription, transcription_rna, differentiation, development	transcription process	1.5496	over-expressed: 4 down-expressed: 20
5	31	mitotic, cell_cycle, cycle, g, cell, apc, transition, apc_c, c, g_transition	cell cycle regulation	−2.5362	over-expressed: 28 down-expressed: 3
6	16	golgi, transport, er, golgi_er, retrograde, vesicle, vesicle_mediated, mediated_transport, mediated, traffic	golgi apparatus related	−0.9197	over-expressed: 11 down-expressed: 5
7	4	biological_process, biological, process	biological processes	2.7369	over-expressed: 0 down-expressed: 4

**Table 3:** Top 15 miRNA genes and DNA methylation loci from the mass MCFS-ID experiments.

miRNA Gene	Freq	Sum RI	Mean RI	MCFS-ID Rank		DNA Methylation	Freq	Sum RI	Mean RI	MCFS-ID Rank
hsa_mir_139	73	65.628	0.899	1		cg07267550	7	3.044	0.435	1234
hsa_mir_141	73	38.519	0.528	10		cg00914963	7	3.036	0.434	2048
hsa_mir_10b	73	36.690	0.503	2		cg19533977	7	3.024	0.432	25
hsa_mir_183	73	33.020	0.452	4		cg08113562	7	2.714	0.388	12,061
hsa_mir_140	73	32.825	0.450	11		cg17901038	7	2.620	0.374	186
hsa_mir_200a	73	31.182	0.427	15		cg18253799	7	2.584	0.369	2217
hsa_mir_96	73	28.417	0.389	8		cg20417953	7	2.504	0.358	6490
hsa_mir_429	73	25.452	0.349	20		cg20524128	7	2.488	0.355	2066
hsa_mir_204	73	23.546	0.323	12		cg10520594	7	2.393	0.342	4407
hsa_mir_99a	73	23.041	0.316	6		cg20701457	7	2.159	0.308	1487
hsa_mir_592	73	22.352	0.306	16		cg16009970	7	2.090	0.299	13,163
hsa_mir_378	73	22.320	0.306	38		cg06976025	7	2.012	0.287	3878
hsa_mir_145	73	22.218	0.304	5		cg15601264	7	1.993	0.285	188
hsa_mir_21	73	21.700	0.297	3		cg22608492	7	1.932	0.276	151
hsa_let_7c	73	21.686	0.297	13		cg11441693	7	1.913	0.273	12,369

**Table 4:** Input data description.

Data Type	Unit of Measurement	Number of Total Samples	Number of Normal Samples	Number of Features
mRNA expression	reads per kilobase million	867	99	20,524
DNA methylation	beta-value	870	97	396,065
miRNA expressions	reads per million miRNA mapped	832	86	897

## Data Availability

Data is contained within the article, Supplementary Materials and TCGA publicly available database.

## Supplementary Materials

The following supporting information can be downloaded at https://www.mdpi.com/article/10.3390/ijms26146558/s1.

## Abbreviations

The following abbreviations are used in this manuscript:

ChIA-PET	Chromatin Interaction Analysis by Paired-End Tag Sequencing
CpG	Cytosine Phosphate Guanine
CpGI	Cpg Island
DEG	Differentially Expressed Gene
DMS	Differentially Methylated Sites
DScore	Drug Score
GScore	Gene Score
GO BP	Gene Ontology Biological Processes
H3K27ac	Acetylation of the Lysine 27 of the Histone H3 Protein
HOCOMOCO	Homo Sapiens Comprehensive Model Collection
KEGG	Kyoto Encyclopedia of Genes and Genomes
log_2_FC	Log_2_ Fold Change
MCFS-ID	Monte Carlo Feature Selection and Interdependencies Discovery
NLP	Natural Language Processing Methods
NCBI	National Center for Biotechnology Information
PCHi-C	Promoter Capture Hi-C
PWM	Position Weighted Matrix
RF	Random Forest
RI	Relative Importance
SM	Spring Model
SVM	Support Vector Machine
TCGA	The Cancer Genome Atlas
TF	Transcription Factor
TF-IDF	Term Frequency-Inverse Document Frequency
TFBS	Transcription Factor Binding Site
TSS	Transcription Start Site
wAcc	Weighted Accuracy
WHO	World Health Organization
