# Supplementary material for: Unveiling Epigenetic Regulatory Elements Associated with Breast Cancer Development
Source: Int J Mol Sci. 2025 Jul 8;26(14):6558. doi: 10.3390/ijms26146558 (PMC12295874; doi:10.3390/ijms26146558)
Supplement: Supplementary file 1 [file ijms-26-06558-s001.zip › ijms-3654605-Figure_S1_IJMS.pdf]

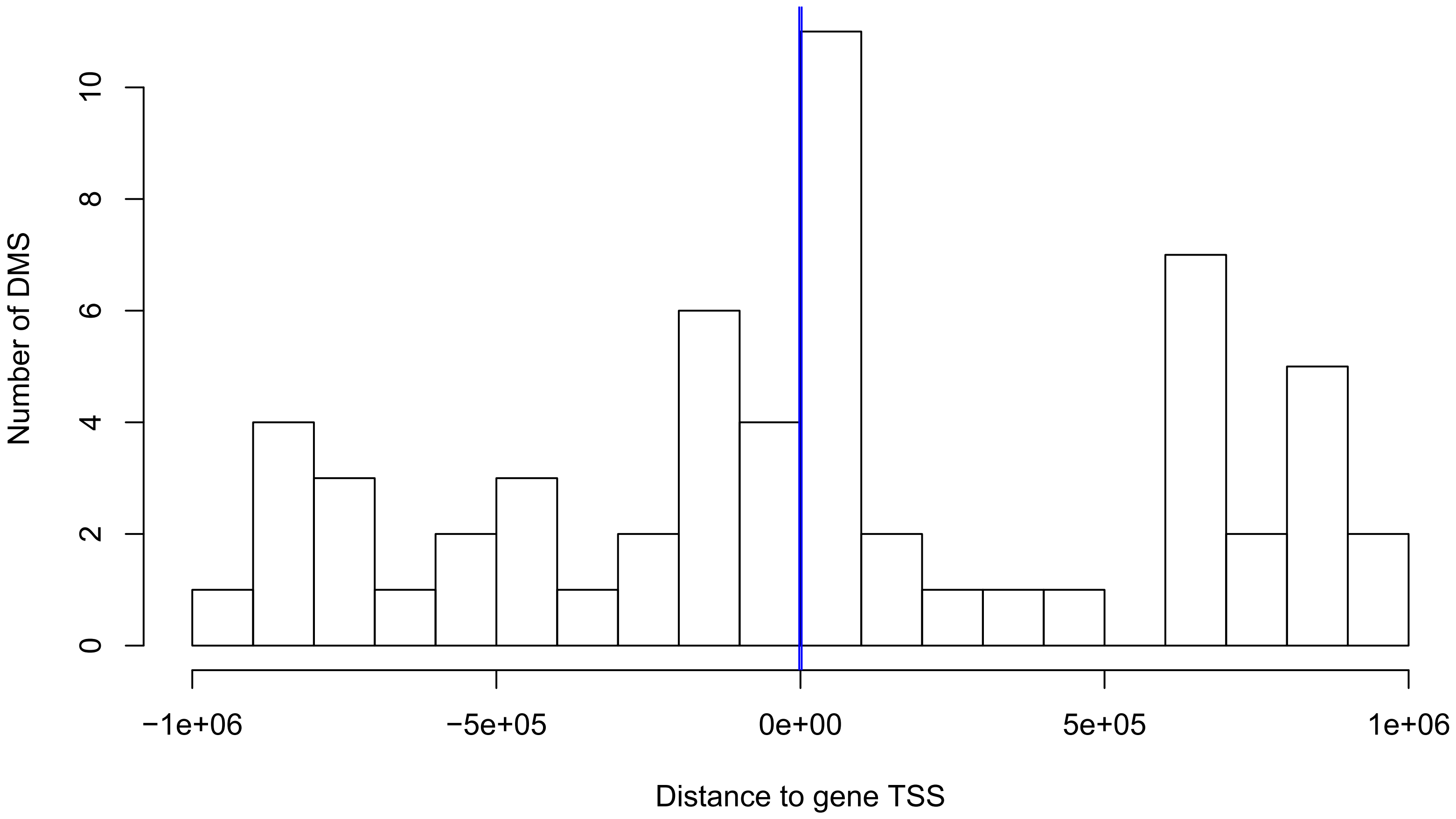

Figure S1. Distances between gene TSS and DNA methylation locus that showed significant correlation
Distribution of distances between gene TSS and DMS that were found to correlate significantly (Spearman correlation, FDR < 0.05, |*rho*| ≥ 0.6). Correlation was performed between gene expression and DMSs located 1Mbp from a gene TSS. The blue line indicated the promoter region ± 2000bp from a TSS.
