# Supplementary material for: Unveiling Epigenetic Regulatory Elements Associated with Breast Cancer Development
Source: Int J Mol Sci. 2025 Jul 8;26(14):6558. doi: 10.3390/ijms26146558 (PMC12295874; doi:10.3390/ijms26146558)
Supplement: Supplementary file 1 [file ijms-26-06558-s001.zip › ijms-3654605-Figure_S2_IJMS.pdf]

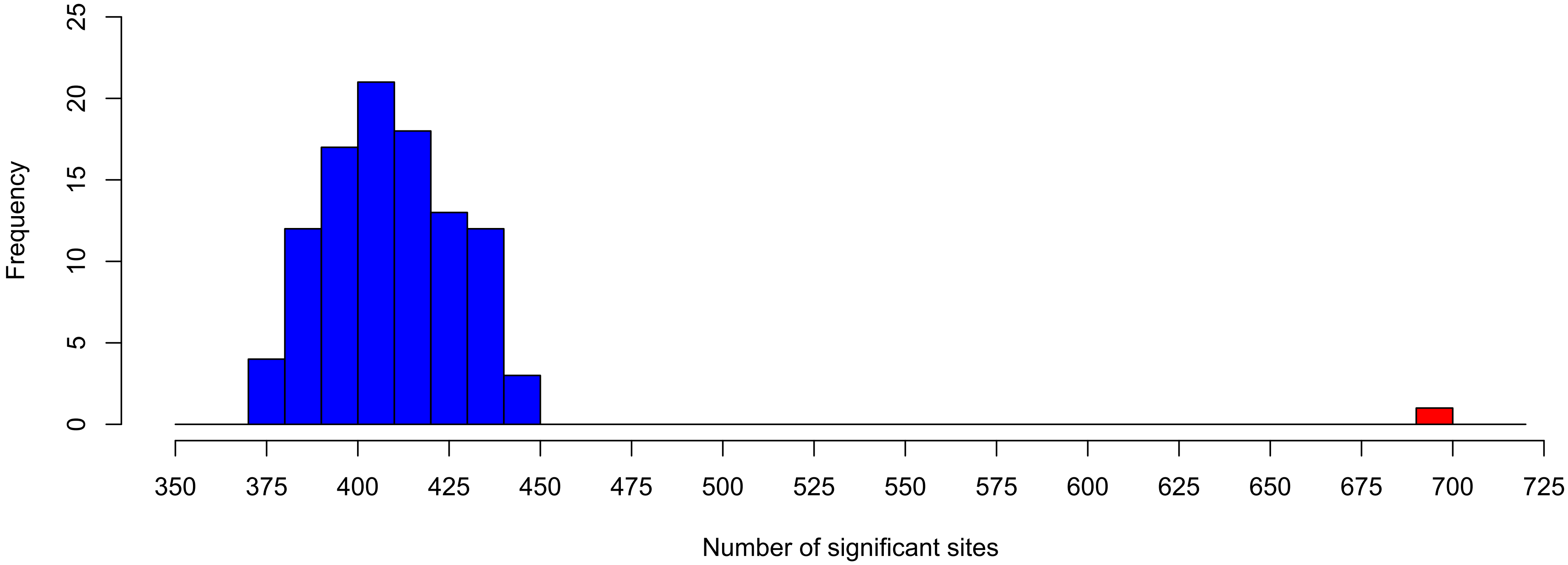

Figure S2. Distribution of the number of sites that had a significant impact on the patient survival
Blue - distribution of significant sites from 2006 sites selected randomly using a bootstrapping technique (sampling 100 times); red - number of significant sites from a set of DMS.
