# Supplementary material for: Unveiling Epigenetic Regulatory Elements Associated with Breast Cancer Development
Source: Int J Mol Sci. 2025 Jul 8;26(14):6558. doi: 10.3390/ijms26146558 (PMC12295874; doi:10.3390/ijms26146558)
Supplement: Supplementary file 1 [file ijms-26-06558-s001.zip › ijms-3654605-Figure_S7_IJMS.pdf]

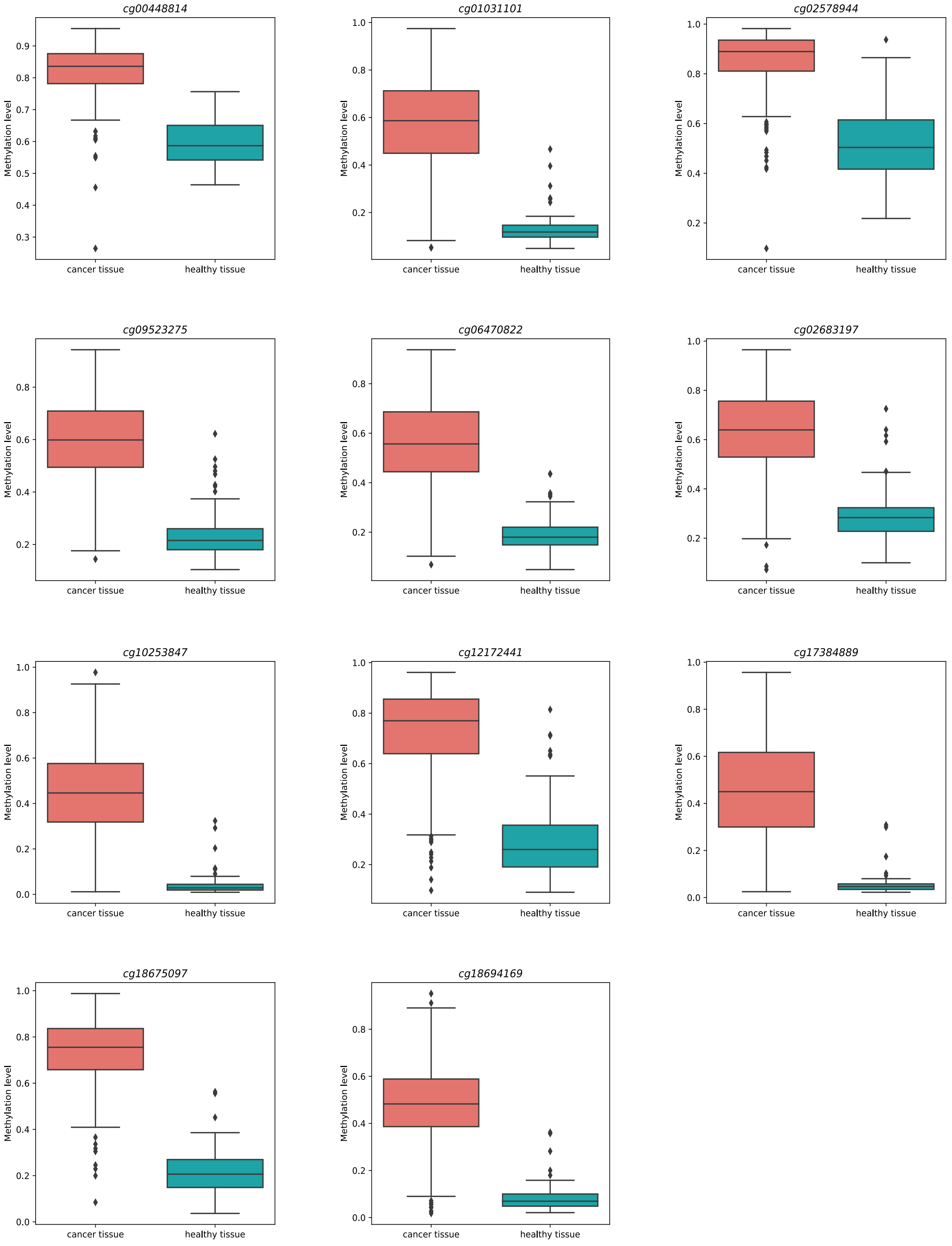

Figure S7. Methylation profiles of significant methylations around the *NKAPL* gene
Methylation profiles of significant methylations around the *NKAPL* gene for cancer tissue (MCF-7) in red and healthy tissue (MCF-10A) in blue.
