# Supplementary material for: Unveiling Epigenetic Regulatory Elements Associated with Breast Cancer Development
Source: Int J Mol Sci. 2025 Jul 8;26(14):6558. doi: 10.3390/ijms26146558 (PMC12295874; doi:10.3390/ijms26146558)
Supplement: Supplementary file 1 [file ijms-26-06558-s001.zip › ijms-36546050-Figure_S3_IJMS.pdf]

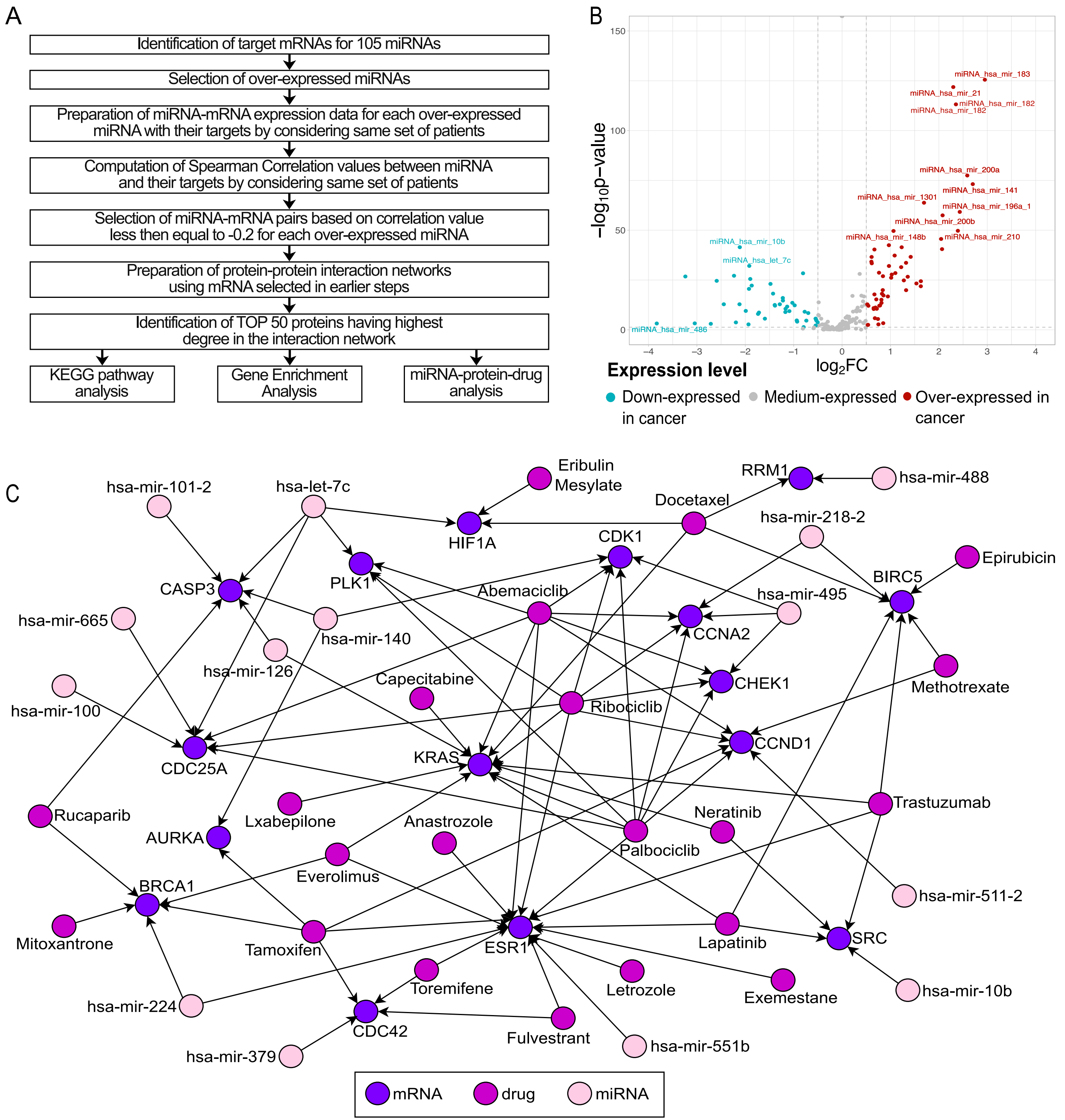

Figure S3. Characterization of differential miRNA genes

(A) Pipeline of miRNAs with suppressive impact on mRNA (B) Volcano plot showing differential miRNAs (C) miRNA-protein-drug network.
