# Supplementary material for: Unveiling Epigenetic Regulatory Elements Associated with Breast Cancer Development
Source: Int J Mol Sci. 2025 Jul 8;26(14):6558. doi: 10.3390/ijms26146558 (PMC12295874; doi:10.3390/ijms26146558)
Supplement: Supplementary file 1 [file ijms-26-06558-s001.zip › ijms-36546050-Figure_S4_IJMS.pdf]

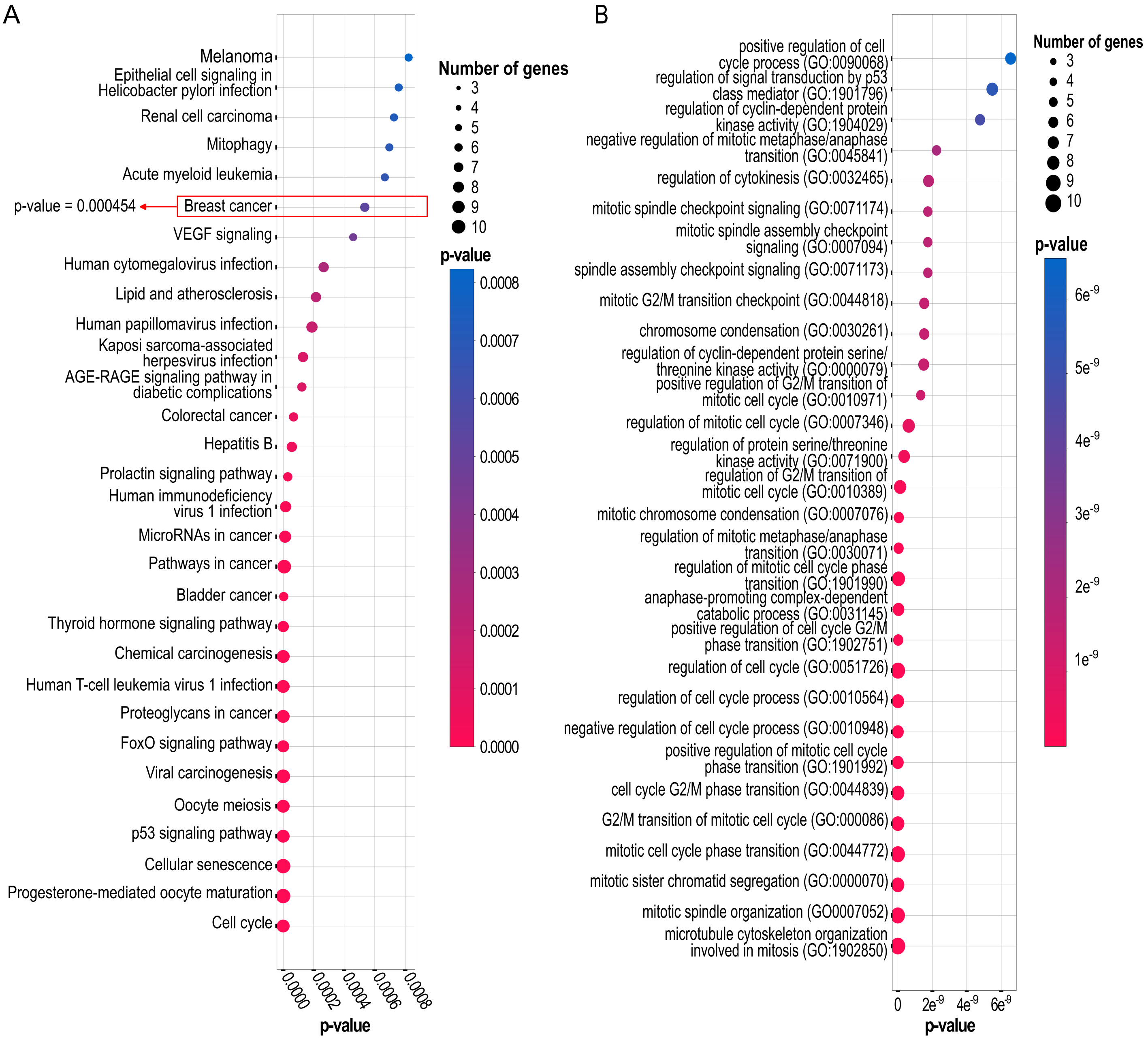

Figure S4. Functional analysis of the over-expressed genes encoding proteins with the highest number of interaction

Functional analysis of the over-expressed genes encoding 50 proteins with the highest number of interaction network (A) KEGG pathway analysis (B) GO BP analysis.
