# Supplementary material for: Unveiling Epigenetic Regulatory Elements Associated with Breast Cancer Development
Source: Int J Mol Sci. 2025 Jul 8;26(14):6558. doi: 10.3390/ijms26146558 (PMC12295874; doi:10.3390/ijms26146558)
Supplement: Supplementary file 1 [file ijms-26-06558-s001.zip › ijms-36546050-Figure_S5_IJMS.pdf]

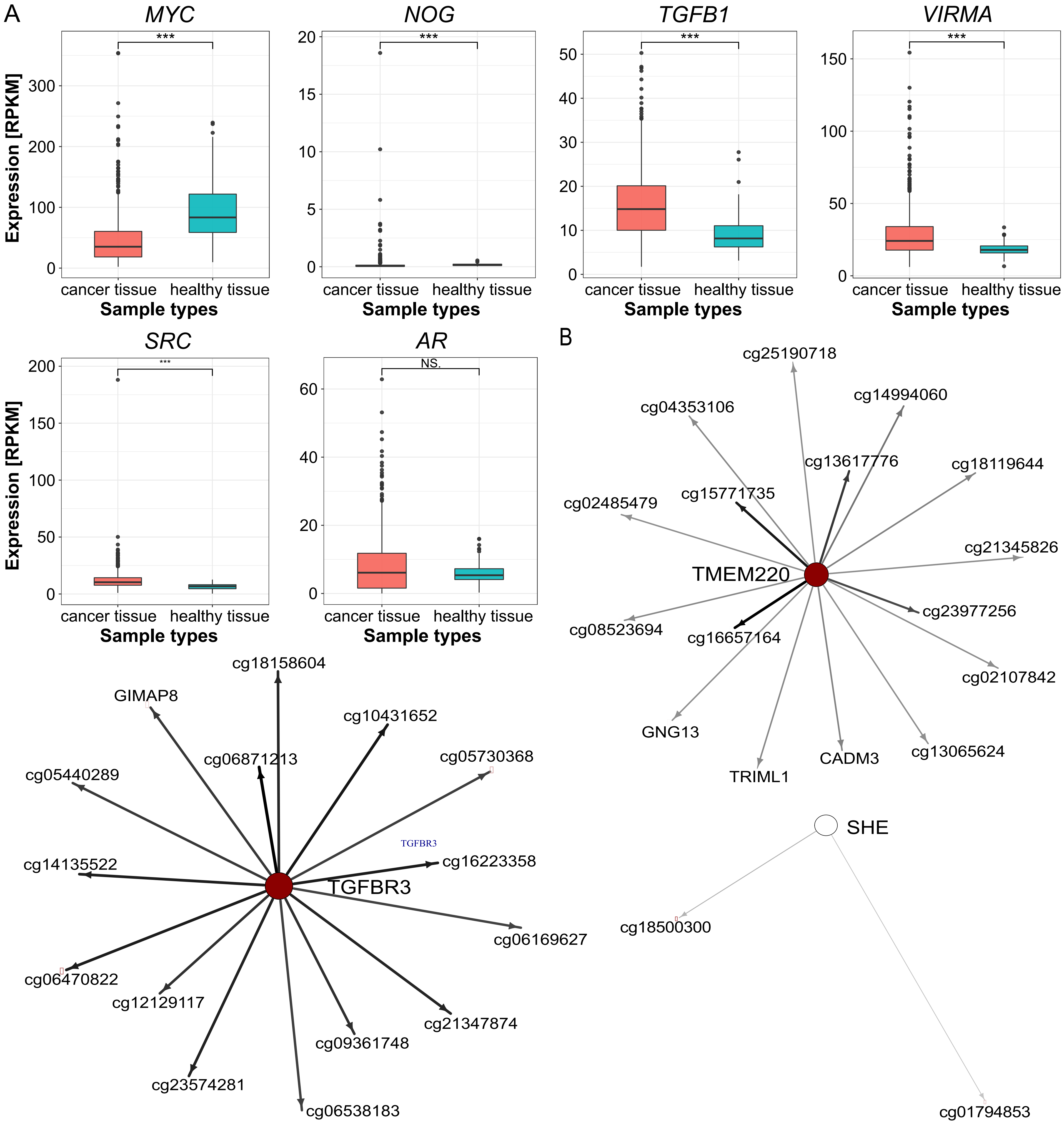

Figure S5. Additional profiles for gene expressions in regulatory networks and ID-Graphs

Additional profiles for genes in regulatory networks (A) Expression profiles of genes with the largest number of putative interactions in gene-gene interactions network. Wilcoxon test was used to compare significance of expression changes between them and *** means p-value≤0.001. (B) Interaction graphs (ID-Graphs) obtained from MCFS-ID for three target genes whose linear models reached statistical significance p≤0.05 and R2>0.5 [TMEM220, TGFBR3, SHE].
