# Supplementary material for: Unveiling Epigenetic Regulatory Elements Associated with Breast Cancer Development
Source: Int J Mol Sci. 2025 Jul 8;26(14):6558. doi: 10.3390/ijms26146558 (PMC12295874; doi:10.3390/ijms26146558)
Supplement: Supplementary file 1 [file ijms-26-06558-s001.zip › ijms-36546050-Figure_S6_IJMS.pdf]

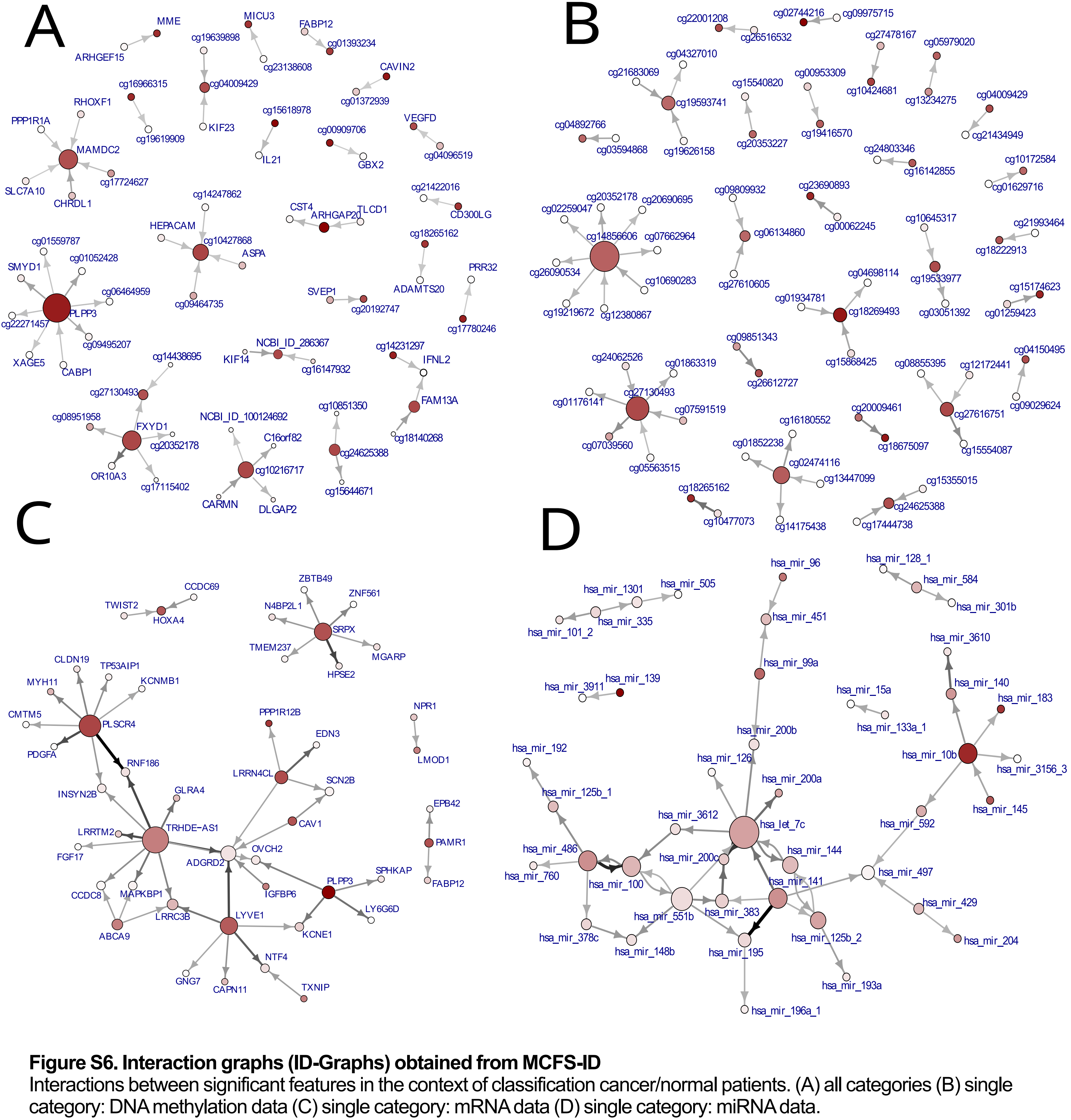

Figure S6. Interaction graphs (ID-Graphs) obtained from MCFS-ID

Interactions between significant features in the context of classification cancer/normal patients. (A) all categories (B) single category: DNA methylation data (C) single category: mRNA data (D) single category: miRNA data.
